# PIEZO1 is a distal nephron mechanosensor and is required for flow-induced K^+^ secretion

**DOI:** 10.1172/JCI174806

**Published:** 2024-03-01

**Authors:** Rolando Carrisoza-Gaytan, Stephanie M. Mutchler, Francisco Carattino, Joanne Soong, Marianela G. Dalghi, Peng Wu, WenHui Wang, Gerard Apodaca, Lisa M. Satlin, Thomas R. Kleyman

**Affiliations:** 1 Department of Pediatrics, Icahn School of Medicine at Mount Sinai, New York, New York, USA.; 2 Department of Medicine, University of Pittsburgh, Pittsburgh, Pennsylvania, USA.; 3 Department of Nephrology, The First Affiliated Hospital of Zhengzhou University, Zhengzhou, China.; 4 Department of Pharmacology, New York Medical College, Valhalla, New York, USA.; 5 Department of Cell Biology and; 6 Department of Pharmacology and Chemical Biology, University of Pittsburgh, Pittsburgh, Pennsylvania, USA.

**Keywords:** Cell biology, Nephrology, Calcium channels, Epithelial transport of ions and water, Potassium channels

## Abstract

Ca^2+^-activated BK channels in renal intercalated cells (ICs) mediate luminal flow–induced K^+^ secretion (FIKS), but how ICs sense increased flow remains uncertain. We examined whether PIEZO1, a mechanosensitive Ca^2+^-permeable channel expressed in the basolateral membranes of ICs, is required for FIKS. In isolated cortical collecting ducts (CCDs), the mechanosensitive cation-selective channel inhibitor GsMTx4 dampened flow-induced increases in intracellular Ca^2+^ concentration ([Ca^2+^]_i_), whereas the PIEZO1 activator Yoda1 increased [Ca^2+^]_i_ and BK channel activity. CCDs from mice fed a high-K^+^ (HK) diet exhibited a greater Yoda1-dependent increase in [Ca^2+^]_i_ than CCDs from mice fed a control K^+^ diet. ICs in CCDs isolated from mice with a targeted gene deletion of *Piezo1* in ICs (IC-*Piezo1*-KO) exhibited a blunted [Ca^2+^]_i_ response to Yoda1 or increased flow, with an associated loss of FIKS in CCDs. Male IC-*Piezo1*-KO mice selectively exhibited an increased blood [K^+^] in response to an oral K^+^ bolus and blunted urinary K^+^ excretion following a volume challenge. Whole-cell expression of BKα subunit was reduced in ICs of IC-*Piezo1*-KO mice fed an HK diet. We conclude that PIEZO1 mediates flow-induced basolateral Ca^2+^ entry into ICs, is upregulated in the CCD in response to an HK diet, and is necessary for FIKS.

## Abstract 2

PIEZO1, a mechanosensitive Ca^2+^ permeable channel expressed in basolateral membranes of intercalated cells, functions as a mechanosensor and, by sensing wall tension, regulates K^+^ secretion.

## Graphical abstract

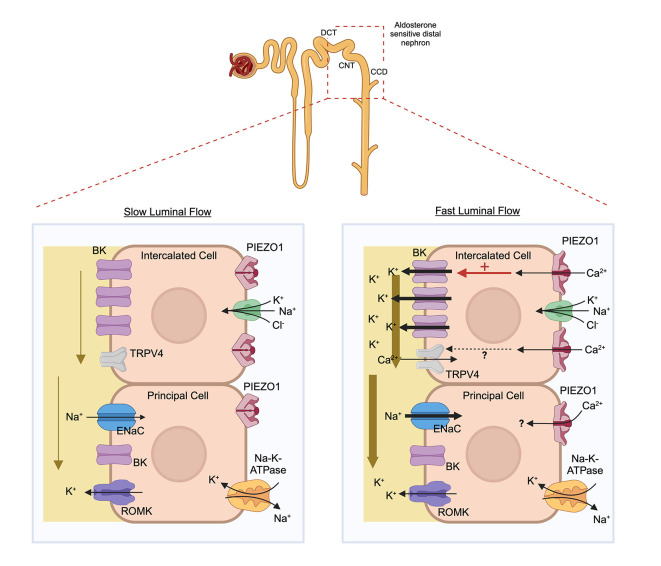

## Introduction

The kidney is subject to variations in tubular (urinary) flow rate that regulate various physiologic functions, including Na^+^ absorption and K^+^ secretion in the aldosterone-sensitive distal nephron (ASDN), composed of the late distal convoluted tubule (DCT2), connecting tubule (CNT), and cortical collecting duct (CCD). Within the ASDN, electrogenic Na^+^ absorption via the apical epithelial Na^+^ channel (ENaC) and its extrusion at the basolateral membrane via the Na^+^-K^+^-ATPase generates a favorable electrochemical gradient for apical K^+^ secretion through apical conducting K^+^ channels. Renal outer medullary K^+^ (ROMK) channels, localized exclusively in principal cells (PCs), mediate constitutive K^+^ secretion, whereas Ca^2+^/stretch-activated large-conductance K^+^ (BK) channels in intercalated cells (ICs) mediate flow-induced K^+^ secretion (FIKS) (1, 2). A high-K^+^ (HK) diet increases apical membrane expression and activity of both BK and ROMK channels (3–5). Loss-of-function mutations in ROMK, as detected in type II Bartter syndrome (6), are associated with urinary K^+^ wasting due to upregulation of BK channel function (7). Similarly, loss-of-function mutations in the BK channel are associated with a compensatory increase in expression of ROMK channels (8).

A rapid increase in tubular fluid flow rate in the ASDN subjects both PCs and ICs therein to 3 types of mechanical forces: hydrostatic pressure, circumferential stretch (in association with an increase in luminal volume), and fluid flow–induced shear or drag forces (9). These hydrodynamic forces are transduced into biphasic increases in intracellular Ca^2+^ concentration ([Ca^2+^]_i_) in both cell populations (1, 2, 9). The flow-induced Ca^2+^ response includes an initial rapid increase in [Ca^2+^]_i_ that peaks within approximately 10 seconds and is due to extracellular Ca^2+^ entry at the basolateral membrane coupled with inositol 1,4,5 triphosphate–dependent (IP_3_-dependent) Ca^2+^-induced Ca^2+^ release (9). The identity of the basolateral entry pathway is unknown, but its sensitivity to flow indicates it may function as a basolateral mechanosensor. The peak [Ca^2+^]_i_ is followed by a decay to a plateau elevation in [Ca^2+^]_i_, sustained throughout a period of high flow, reflecting luminal Ca^2+^ entry that is, in part, mediated by TRPV4 (9–12). The flow-stimulated increase in [Ca^2+^]_i_ leads to the activation of several downstream pathways, including flow-induced K^+^ secretion (FIKS) in ICs (1, 2).

The identity and precise localization of the flow sensors that initiate mechanotransduction in the ASDN remain uncertain. The apical cilia decorating PCs and apical microvilli/microplicae of ICs have been proposed to sense fluid flow (9); however, how these pathways would regulate basolateral Ca^2+^ entry is unknown. One possible candidate is PIEZO1, a mechanoactivated nonselective cation channel (13, 14) that is selectively localized to the basolateral surface of epithelial cells in mouse ASDN (15), although basolateral and apical expression of PIEZO1 was recently reported by Pyrshev et al. (16). Numerous studies report that PIEZO1 and its closely related family member PIEZO2 function as mechanosensors, which are directly activated by cell membrane tension generated in response to membrane stretch or fluid shear stress (17, 18). For example, Piezo2b regulates the light touch response in zebrafish (19). In cultured renal epithelia, PIEZO1 activation via mechanical stretch stimulates cell division (20). Mammalian PIEZO1 plays an essential role in vascular development and endothelial cell orientation in response to fluid shear stress in the mouse embryo (21, 22). Mammalian PIEZO2 is required for touch sensation in skin Merkel cells (23), proprioception (24), and airway stretch and respiration (25). Both PIEZO1 and PIEZO2 contribute to blood pressure homeostasis (26, 27), responses of chondrocytes to compression (28), and mechanotransduction in native bladder umbrella cells (29). These channels are critical in development, as mice with global *Piezo1* or *Piezo2* knockout die during embryogenesis or at birth, respectively (21, 22). Human mutations in PIEZO channels are associated with disorders including xerocytosis, congenital lymphatic dysplasia, distal arthrogryposis, defects in proprioception, bladder dysfunction, and neonatal respiratory insufficiency (24, 30–36).

PIEZO1 is primarily expressed in nonneuronal cells including kidney, bladder, colon, and lung, although recent studies reveal that PIEZO1 is also expressed in sensory neurons (37). In contrast, PIEZO2 is generally expressed in sensory neurons as well as in bladder and colonic epithelial cells (29, 36, 38, 39). Limited information exists regarding the expression and functional roles of PIEZO channels in the kidney. However, quantitative reverse transcription PCR revealed that *Piezo1* message in kidney is much greater than that of *Piezo2* (40, 41) and that *Piezo2* expression in the kidney is relatively low (13). In the adult mouse, the highest levels of *Piezo1* expression are in the outer and inner medullary collecting ducts, with expression also noted in the thick ascending limb, DCT, CNT, and CCD (42). RNA-Seq studies have shown similar expression of *Piezo1* in ICs and PCs (Knepper Epithelial Systems Biology Laboratory database) (43), whereas *Piezo2* was not detected. Low levels of *Piezo1* expression are observed in proximal tubule segments (44), consistent with an absence of PIEZO1 immunolocalization in mouse proximal tubule (15). However, PIEZO1-mediated mechanosensitive ion currents are reported in isolated proximal tubules and proximal tubule cells (41). Few studies have defined a functional role for PIEZO channels in kidney function. However, in response to rehydration following dehydration, mice with Ksp-cadherin Cre-driven deletion of *Piezo1* in the collecting duct, loop of Henle, and distal tubule exhibit delays in urinary dilution and a decrease in the concentration of urinary urea via an unknown pathway (40). A recent study suggested that PIEZO2 has a role in renin release from juxtaglomerular granular cells (45).

We recently reported that PIEZO1 is expressed along the basolateral membranes of ICs and PCs (15), a location ideally suited to sense an increase in wall tension induced by a flow-induced increase in tubule diameter in the CCD (9). We hypothesized that basolateral PIEZO1 channels in ICs function as mechanosensors in the CCD, enabling FIKS by facilitating basolateral Ca^2+^ entry, triggering the requisite increase in [Ca^2+^]_i_ in ICs. We focused this investigation on ICs given that an increase in luminal flow rate in the CCD leads to Ca^2+^-dependent FIKS, mediated by BK channels in this unique cell population. The role of flow-induced increases in [Ca^2+^]_i_ in PC function is unknown. Using pharmacologic and genetic approaches in mice, we show that PIEZO1 mediates flow-induced basolateral Ca^2+^ entry into PCs and ICs, is upregulated in ICs and PCs by an HK diet, and is required for the flow-induced increase in [Ca^2+^]_i_ in ICs and for FIKS. Additionally, male but not female mice lacking PIEZO1 expression in ICs exhibit an exaggerated increase in blood [K^+^] in response to an oral K^+^ load and have a blunted upregulation of K^+^ secretion in response to an acute volume load.

## Results

### A basolateral mechanosensitive channel contributes to flow-induced increases in [Ca^2+^]_i_.

**Figure 1:**
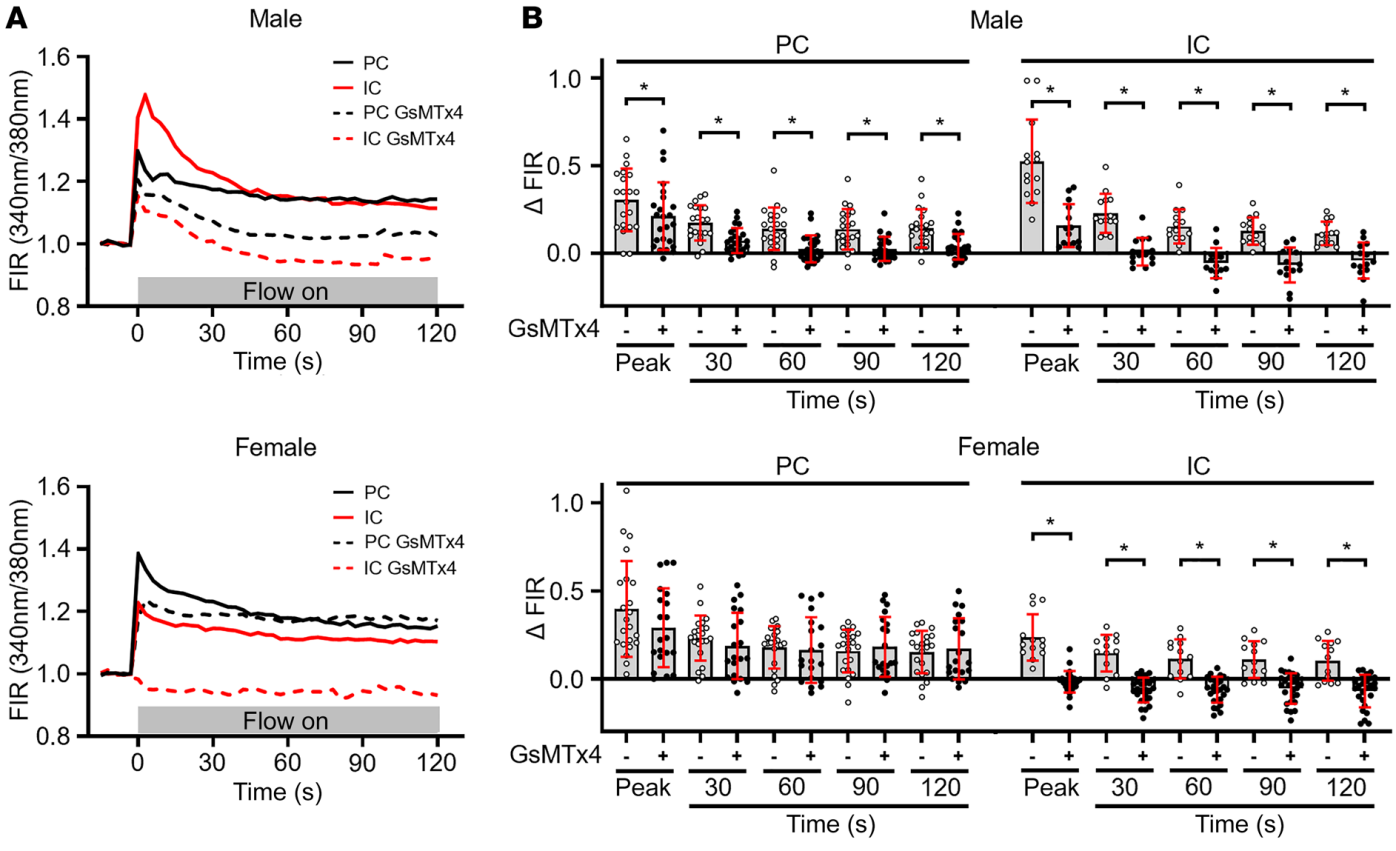
Effect of the PIEZO inhibitor GsMTx4 on luminal flow–induced increases in [Ca^2+^]_i_ in ICs and PCs in microperfused CCDs. (**A**) Representative tracings of fura-2 fluorescence intensity ratios (FIRs), equivalent to [Ca^2+^]_i_, in individual PCs and ICs in fura-2–loaded CCDs subjected to an acute increase in tubular flow rate, isolated from male (top) and female (bottom) C57BL/6J mice. In untreated cells, the increase in luminal flow triggered a biphasic response, including an immediate rapid increase in FIR to a peak value followed by a gradual decay to a plateau elevation in FIR. GsMTx4 (5 μM) dampened the flow-induced increase in [Ca^2+^]_i_. (**B**) Summary graph presenting the change in FIRs from baseline for all PCs and ICs studied and mean ± SD in the absence (open circles) and presence (filled circles) of GsMTx4 added to the bath solution. FIR was determined before and at designated intervals after an acute increase in flow rate. *n* (mice) = 4 male control, 3 male GsMTx4, 3 female control, and 3 female GsMTx4. One CCD was studied per animal, and 8–19 cells studied per CCD. *P < 0.05 vs. presence of GsMTx4 at specific times (seconds), analyzed by 2-tailed unpaired Student’s *t* test.

To determine whether PIEZO channels have a role in flow-dependent intracellular Ca^2+^ signaling in the CCD, we examined the effect of the L-enantiomer of GsMTx4, a peptide inhibitor of cation-selective mechanosensitive channels including PIEZO1 but not TRPV4 channels (46–48). ICs and PCs in fura-2–loaded microperfused CCDs isolated from wild-type (WT) mice fed a control K^+^ (CK) diet exhibited a biphasic [Ca^2+^]_i_ response to a rapid increase in luminal flow sufficient to increase tubule diameter by 23% ± 3% (Figure 1). Preincubation (15 minutes) of CCDs in a bath containing 5 μM GsMTx4 blunted the luminal flow–induced [Ca^2+^]_i_ increase in ICs and PCs, but in a sex-dependent manner. Specifically, the inhibitor significantly reduced the initial peak response and late-phase plateau elevation in [Ca^2+^]_i_ in female and male ICs. However, only male PCs exhibited a significantly blunted initial peak and late-phase plateau elevation in [Ca^2+^]_i_. These observations support the hypothesis that a basolateral mechanosensitive channel, possibly PIEZO1, contributes to flow-induced [Ca^2+^]_i_ transients in ICs.

### Yoda1 increases [Ca^2+^]_i_ and activates BK channels in CCDs.

**Figure 2:**
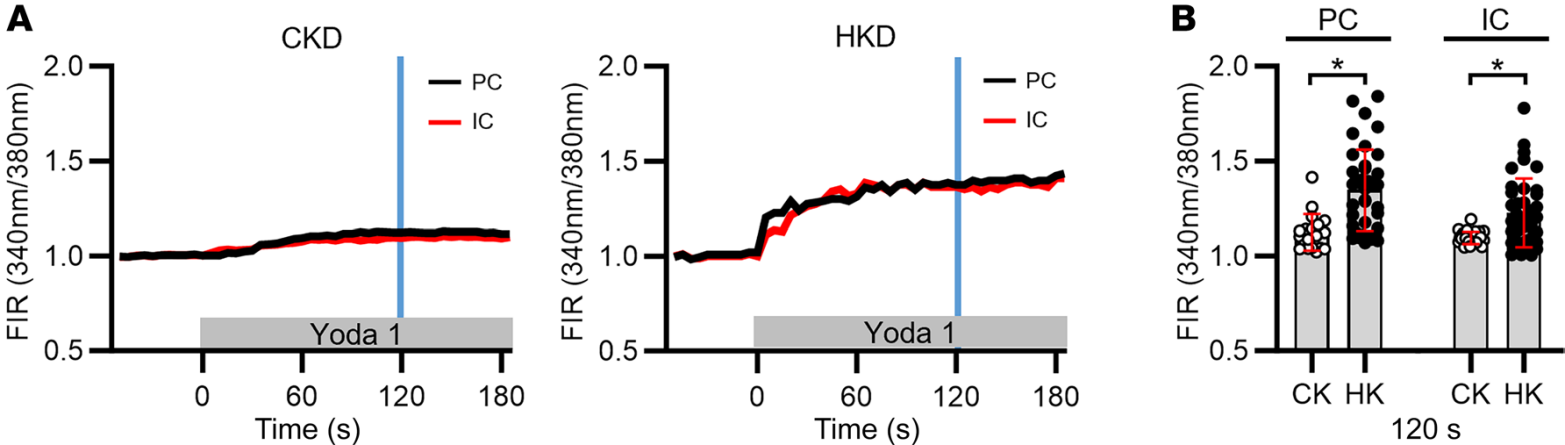
Effect of Yoda1 on [Ca^2+^]_i_ in CCDs isolated from C57BL/6J mice fed a CK or HK diet for 10 days, and microperfused at a very slow flow rate. (**A**) Representative traces of the [Ca^2+^]_i_ responses recorded in individual ICs (red) and PCs (black) before and after addition of Yoda1 (1 μM) to the bath solution. (**B**) Summary graph showing individual data points and mean ± SD for FIRs, normalized to the baseline FIR, in PCs and ICs measured at 120 seconds (identified by the blue vertical lines in **A**) after exposure to Yoda1, comparing data from mice fed a CK or HK diet. No significant differences in the response in FIR were detected between individual cell types for a given diet. An HK diet enhanced the Yoda1 responses in both cell types. *n* = 3 male mice per group; 1 CCD studied per animal and 14–28 cells studied per CCD. *P < 0.05 CK vs. HK diet for a given cell type, analyzed by 2-tailed unpaired Student’s *t* test.

As further evidence that PIEZO1 activation directly leads to an increase in [Ca^2+^]_i_, we examined the effect of adding the PIEZO1 activator Yoda1 (1 μM) (14, 49) to the bath of CCDs microperfused at low flow rates. CCDs were isolated from male WT mice fed a CK or an HK diet. An HK diet leads to an increase in apical immunoreactive BKα, the pore-forming subunit of the BK channel, and activity of conducting BK channels in mouse ICs and PCs (1, 4). In CCDs isolated from CK-fed mice, Yoda1 application led to a gradual increase in [Ca^2+^]_i_, which plateaued after about 20 seconds in both ICs and PCs (Figure 2, A and B). This increase in [Ca^2+^]_i_ was significantly more pronounced in animals fed an HK diet when measured at 120 seconds (Figure 2, A and B). Yoda1 concentrations of 1 or 5 μM were not associated with any apparent cell toxicity as evidenced by trypan blue exclusion (data not shown).

**Figure 3:**
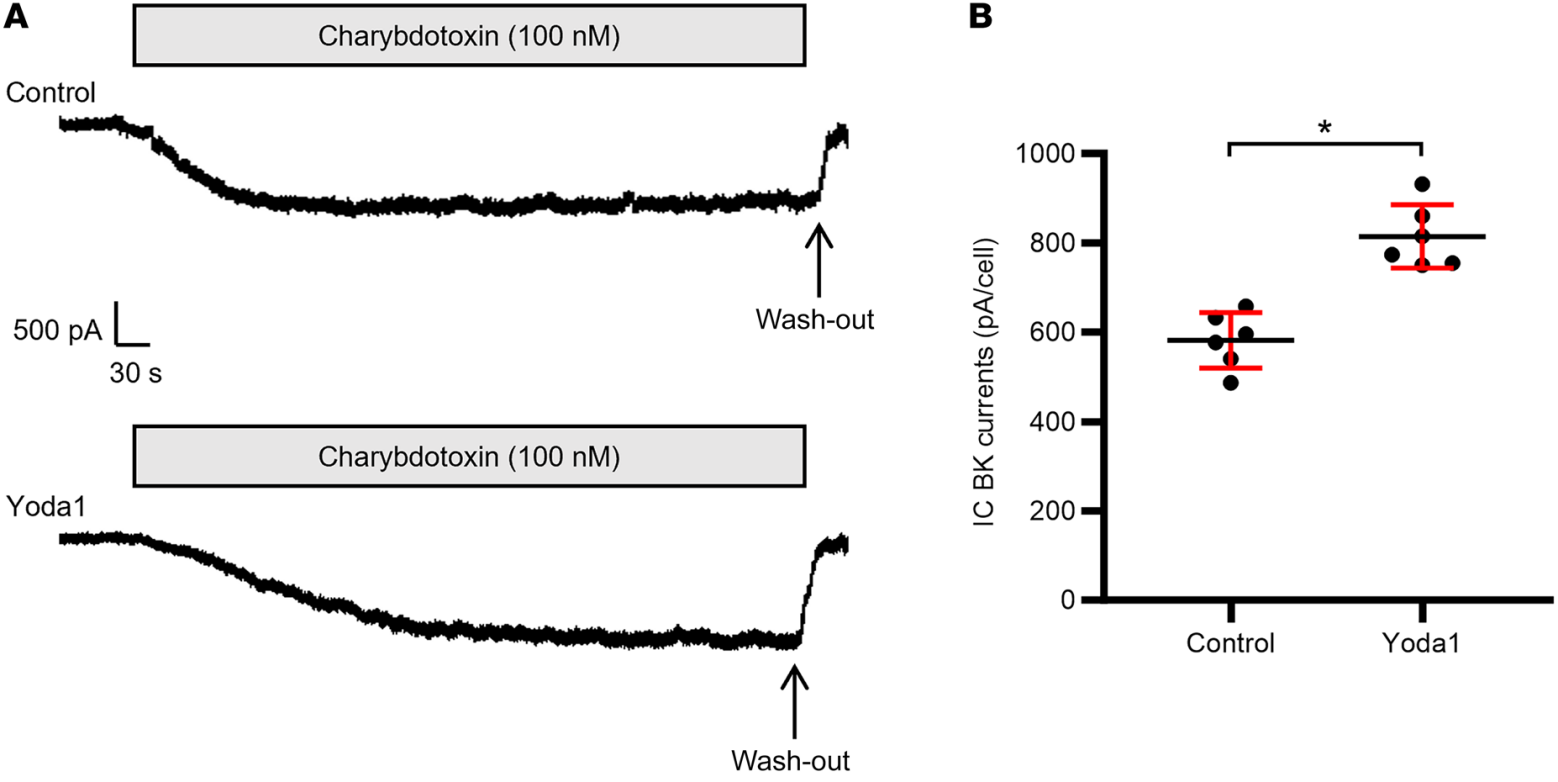
Effect of Yoda1 on charybdotoxin-sensitive BK channel currents in ICs in C57BL/6J mice fed a CK diet. Perforated whole-cell patch recordings were performed in cells clamped at +60 mV. The composition of the bath and pipette solutions, which both contained 130 mM K^+^-gluconate, is given in Supplemental Methods. (**A**) Representative current tracings in ICs in the absence (top) or presence (bottom) of 5 μM Yoda1. (**B**) Summary graph showing individual data points and mean ± SD for charybdotoxin-sensitive current density in control and Yoda1-treated ICs, normalized to the average whole-cell membrane capacitance of 13 pF/cell. Currents in ICs treated with Yoda1 (814 ± 71 pA; *n* = 6 cells) were greater than those in the absence (581 ± 62 pA; *n* = 6 cells) of the PIEZO1 activator. *n* = 3 male mice; 4 cells studied per mouse (2 control and 2 Yoda1-treated). *P < 0.001 by 2-tailed unpaired Student’s *t* test.

To test whether the Yoda1-associated increase in [Ca^2+^]_i_ affects BK channel activation, we performed perforated whole-cell recordings of charybdotoxin-sensitive K^+^ currents, mediated by BK channels (2, 50), in ICs clamped at +60 mV. CCDs were isolated from male WT mice (*n* = 3) fed a CK diet and studied in the absence or presence of Yoda1 (5 μM) added to the bath solution (Figure 3). IC BK channel currents, normalized to the average whole-cell capacitance (13 pF/cell), were significantly greater in the presence (814 ± 71 pA) than in the absence (581 ± 62 pA) of Yoda1, demonstrating that pharmacologic activation of PIEZO1 may activate BK channels in the absence of mechanical forces induced by high flow.

### PIEZO1 expression is increased by dietary K^+^ loading.

**Figure 4:**
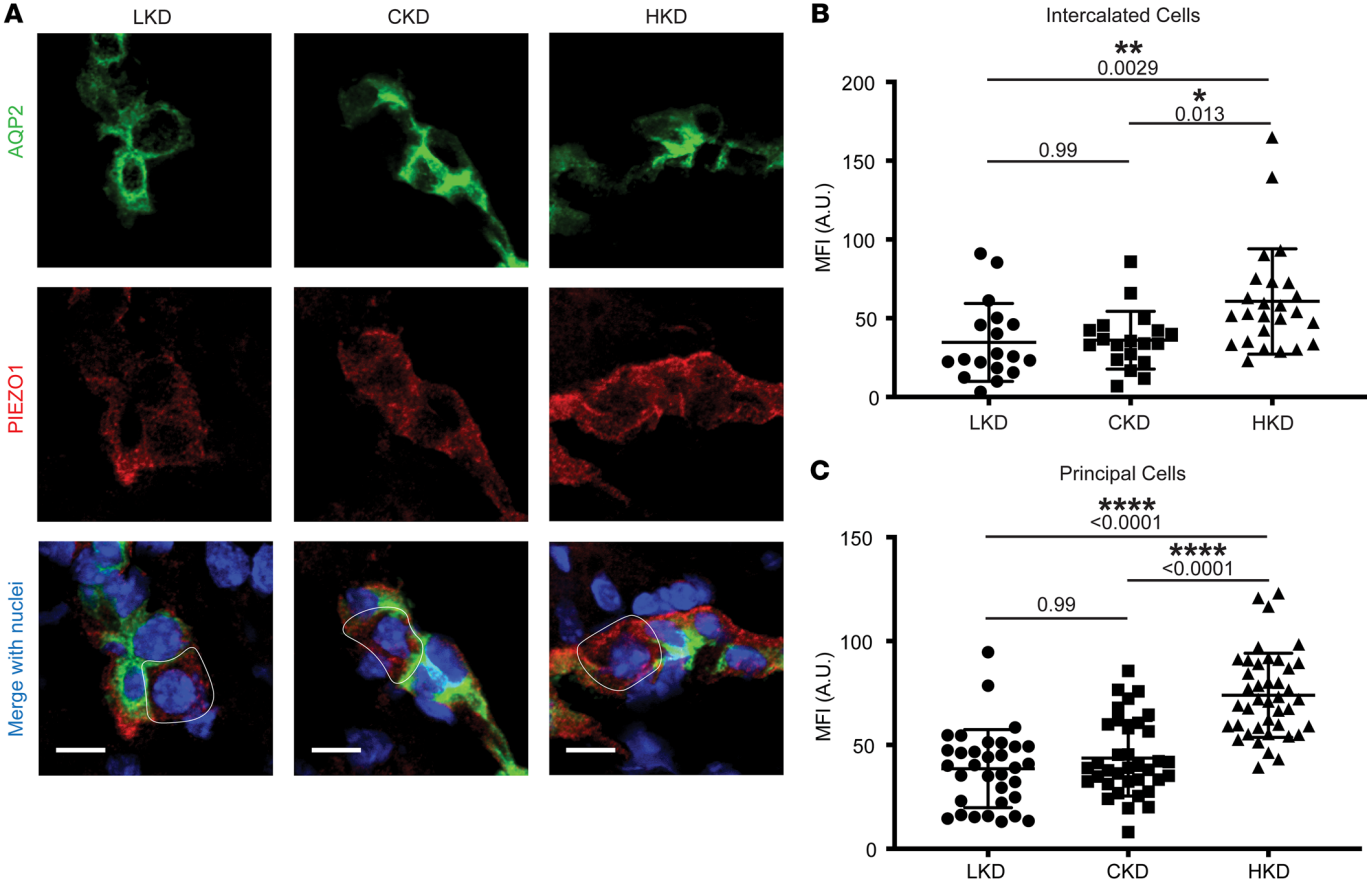
Effect of dietary K^+^ on PIEZO1 expression in *Piezo1^tdT/tdT^* mouse CDs. (**A**) Representative confocal micrographs of cryosections from *Piezo1^tdT/tdT^* mice fed an LK (0.3% NaCl, <0.05% K^+^), CK (0.3% NaCl, 1% K^+^), or HK (0.3% NaCl, 5.2% K^+^ as KCl) diet for 10 days. Sections were colabeled with antibodies directed against AQP2 (green, top) and tdTomato (tdT) (red, middle) to localize endogenous PIEZO1-tdT (15). Merged images (bottom) also show DAPI (nuclei) localization (blue). PCs were identified as AQP2-positive cells, while ICs were identified by their lack of AQP2 expression within the AQP2-positive tubule; representative ICs are outlined by a white line. Scale bars: 10 μm. (**B** and **C**) Quantification of PIEZO1-tdT expression (fluorescent signal) in ICs (**B**) and PCs (**C**) from mice on an LK, CK, or HK diet. A significant increase in PIEZO1-tdT expression was observed in ICs from mice fed an HK diet, compared with mice on an LK or CK diet, with exact *P* values shown for each comparison in the figure (mean ± SD; Kruskal-Wallis test, *n* = 18–25 ICs represented by individual points from *n* = 4 male mice in each group). A significant increase in PIEZO1-tdT expression was also observed in PCs from mice fed an HK diet, compared with mice on an LK or CK diet, with *P* values shown for each comparison in the figure (mean ± SD; Kruskal-Wallis test, *n* = 33–41 PCs represented by individual points from *n* = 4 male mice in each group). *P < 0.05, ** *P* < 0.01, ****P < 0.0001.

Given the exaggerated Yoda1-induced increase in [Ca^2+^]_i_ observed in mice fed an HK compared with a CK diet, we sought to examine whether PIEZO1 protein expression in ICs was regulated by dietary K^+^ intake. As commercially available antibodies are not sufficiently sensitive to detect endogenous PIEZO1 expression in mouse kidney (22), these studies were performed in transgenic PIEZO1–tandem dimer Tomato (tdT) reporter mice as previously described (15). Quantification of PIEZO1-tdT fluorescence intensity in ICs, which do not express aquaporin 2 (AQP2), in CCDs revealed a significant increase in PIEZO1-tdT expression in mice fed an HK diet, compared with mice fed a CK or low-K^+^ (LK) diet (Figure 4). We also found that PIEZO1-tdT fluorescence intensity increased significantly in AQP2-positive PCs in mice fed an HK diet, compared with that observed in mice given a CK or LK diet (Figure 4).

### IC-specific deletion of Piezo1 in ICs.

**Figure 5:**
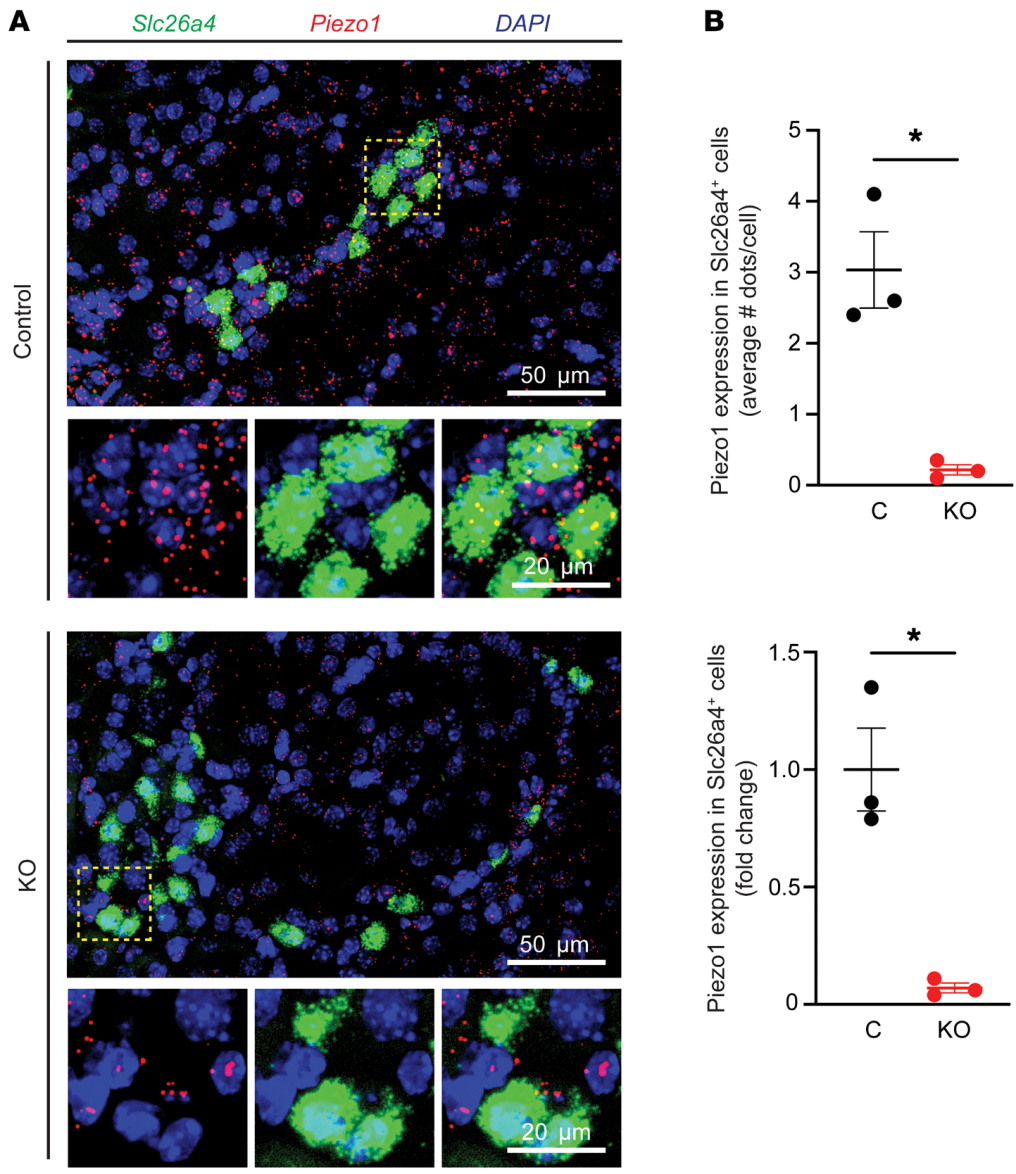
Expression of *Piezo1* mRNA in control and IC-*Piezo1*-KO mouse ICs. (**A**) FISH analysis of *Piezo1* (red) and *Slc26a4* (green) expression in the indicated mouse strains. Nuclei are stained in blue. Scale bars: 50 μm. The area boxed by a yellow dashed line is magnified in the inset; scale bars: 20 μm. (**B**) Levels of *Piezo1* expression in *Slc26a4^+^* cells were quantified using FISH and expressed as the average number of dots per cell (top) or normalized to data obtained for control animals (bottom). Data are mean ± SD (*n* = 3 mice per group) and were tested for normality and analyzed by a 2-tailed unpaired *t* test with Welch’s correction. *P < 0.05.

To better understand the role of PIEZO1 in FIKS, we generated IC-specific *Piezo1*-KO (IC-*Piezo1*-KO) mice and littermate controls. RNAscope (Advanced Cell Diagnostics) was used to confirm a greater than 90% decrease in *Piezo1* expression in ICs expressing *Slc26a4* (pendrin; a marker of type B ICs) in these KO mice (Figure 5). IC-*Piezo1*-KO mice were born at a frequency of 20% female and 25% male, gained weight and developed normally, were fertile, and had no gross morphologic abnormalities. Male and female IC-*Piezo1*-KO animals with free access to food and water showed no significant differences in body weight, 24-hour urinary volume, or urinary electrolyte excretion as compared with their control counterparts (Table 1). In addition, neither males nor females had differences in blood electrolyte composition or aldosterone levels between control and KO animals when fed either a CK or an HK diet when routinely measured in the morning (Table 1).

### [Ca^2+^]_i_ responses to luminal flow and Yoda1 are blunted in ICs from IC-Piezo1-KO mice.

**Figure 6:**
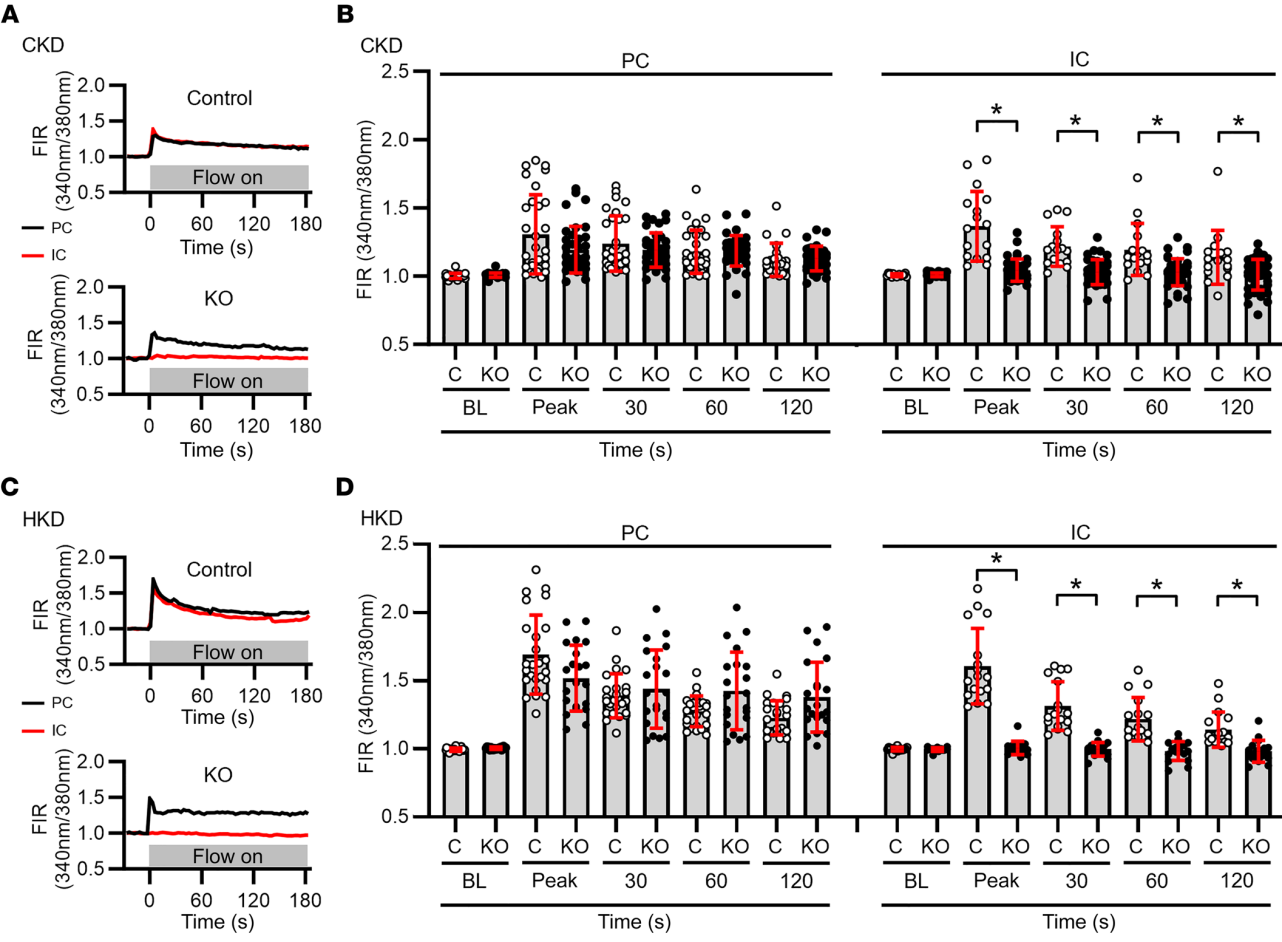
Effect of targeted deletion of *Piezo1* in ICs on flow-induced increases in [Ca^2+^]_i_ in ICs and PCs in microperfused CCDs isolated from IC-*Piezo1*-KO and littermate control mice fed a CK or HK diet. (**A** and **C**) Representative tracings of individual PCs and ICs in CCDs from control littermates and KO mice fed a CK (**A**) or HK (**C**) diet, before and after an acute increase in tubular flow rate. The FIRs in ICs (red) and PCs (black) in fura-2–loaded CCDs were normalized to the FIR measured immediately before the increase in flow rate. An acute increase in luminal flow rate led to a typical biphasic response in ICs and PCs in control CCDs and in PCs from KO CCDs, but not in ICs in KO CCDs. (**B** and **D**) Individual FIRs and means ± SD in PCs and ICs in CCDs isolated from CK-fed (**B**) and HK-fed (**D**) mice are shown at specified times after initiation of high luminal flow. The flow-induced increases in [Ca^2+^]_i_ were virtually absent in ICs in CCDs from KO mice. BL, baseline. For CK diet, *n* (mice and CCDs) = 3 control (open circles, all male) and 5 KO (filled circles, 3 male, 2 female). For HK diet, *n* (mice and CCDs) = 4 control (open circles, 2 male, 2 female) and 4 KO (filled circles, 2 male, 2 female). Eight to 21 cells were studied in each CCD. *P ≤ 0.001, KO vs. control, analyzed by 2-tailed unpaired Student’s *t* test.

We next examined flow-induced [Ca^2+^]_i_ responses in control and IC-*Piezo1*-KO mice fed a CK or HK diet. In microperfused CCDs isolated from control mice, an acute increase in luminal flow led to a typical rapid increase in [Ca^2+^]_i_ in both PCs and ICs (Figure 6), followed by a gradual decay to a sustained plateau level that was above baseline. In tubules isolated from IC-*Piezo1*-KO mice, an increase in flow elicited the expected increase in [Ca^2+^]_i_ in PCs, but was absent in ICs (Figure 6). Interestingly, in ICs, both the early peak increase in [Ca^2+^]_i_ and the sustained plateau elevation in [Ca^2+^]_i_ were lost in KO mice, indicating that PIEZO1 contributes not only to the rapid influx of Ca^2+^ with the initiation of flow, but also to the sustained elevation in [Ca^2+^]_i_ in the continued presence of luminal flow.

**Figure 7:**
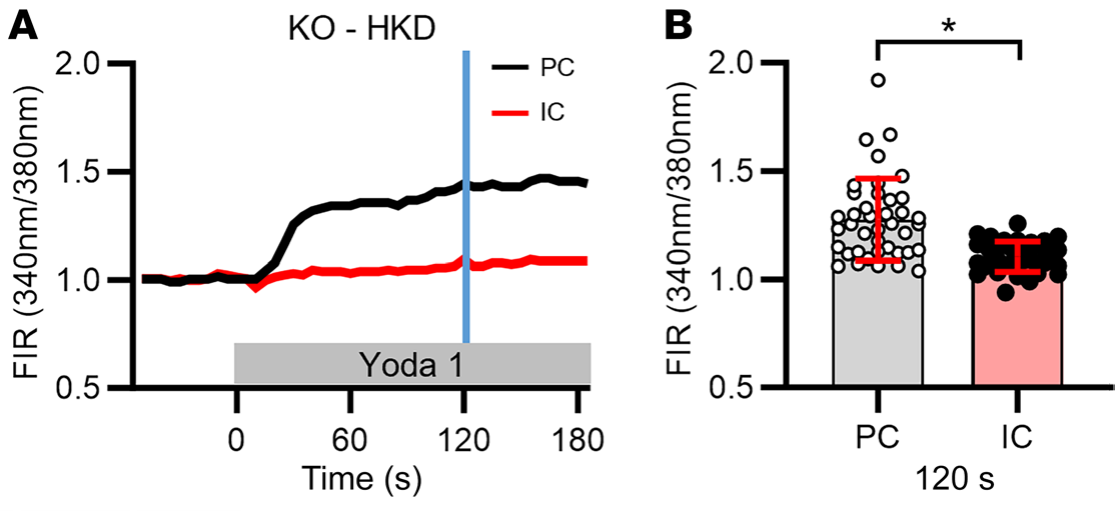
Effect of Yoda1 on [Ca^2+^]_i_ in CCDs isolated from IC-*Piezo1*-KO mice fed an HK diet and microperfused at a very slow flow rate. (**A**) Representative traces of the [Ca^2+^]_i_ responses recorded in an individual IC (red) and PC (black) before and after the addition of Yoda1 (1 μM) to the bath solution. (**B**) Summary graph showing individual data points and mean ± SD for FIRs, normalized to the baseline FIR, in PCs and ICs measured at 120 seconds (indicated by blue vertical line in **A**) after exposure to Yoda1. Yoda1 led to an increase in [Ca^2+^]_i_ in PCs but the IC response was absent in the KO mice. *n* = 3 male mice per group; 1 CCD studied per animal and 25–31 cells studied per CCD. *P < 0.05, data were analyzed by 2-tailed unpaired Student’s *t* test.

ICs in CCDs isolated from HK-fed IC-*Piezo1*-KO mice also exhibited a markedly dampened increase in [Ca^2+^]_i_ in response to basolateral application of the PIEZO1 activator Yoda1 (1 μM) (Figure 7) compared with their control counterparts (Figure 2). The increase in [Ca^2+^]_i_ elicited by Yoda1 in ICs from IC-*Piezo1*-KO animals was also significantly less than that observed in adjacent PCs in the same tubules (Figure 7).

### In vitro microperfused CCDs isolated from IC-Piezo1-KO mice fail to exhibit FIKS.

**Figure 8:**
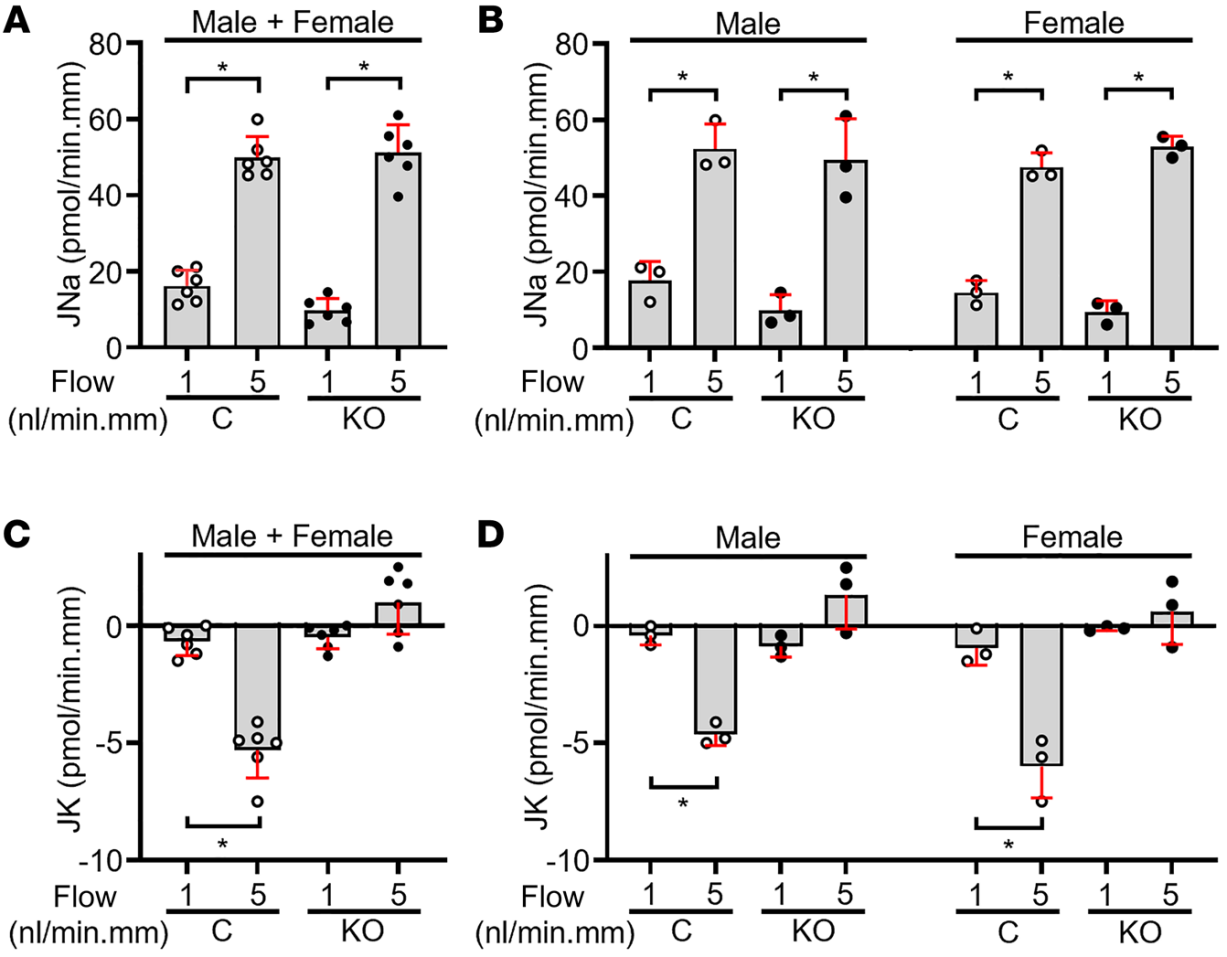
Basal and flow-induced JNa and JK in microperfused CCDs isolated from HK-fed IC-*Piezo1*-KO and control floxed mice. (**A** and **C**) In 6 CCDs from control mice (open circles, 3 male and 3 female), a 5-fold increase in tubular fluid flow rate from 1 (slow) to 5 (fast) nL/min per mm was associated with a significant increase in JNa (**A**) and JK (**C**). (**A** and **B**) Basal JNa and flow-stimulated JNa were similar in CCDs from the controls compared with 6 KO mice (filled circles, 3 male and 3 female). (**C** and **D**) However, FIKS was absent in CCDs from KO male and female mice. Sex-specific transport data are shown in **B** and **D**. Data are represented as mean ± SD; *P < 0.05 vs. JNa or JK at 1 nL/min per mm in the same tubules, analyzed by 2-tailed paired Student’s *t* test. One CCD was studied per mouse.

The impact of the IC-specific disruption of *Piezo1* on basal and flow-induced net Na^+^ absorption (JNa) and K^+^ secretion (JK) was studied in microperfused CCDs isolated from male and female mice fed an HK diet to maximize BK channel expression (1, 3). In CCDs from control mice, an increase in tubular flow rate from 1 (slow) to 5 (fast) nL/min per mm was associated with a significant increase in JNa (from 16.2 ± 4.1 to 49.9 ± 5.5 pmol/min per mm; *P* ≤ 0.001) and JK (from –0.7 ± 0.2 to –5.3 ± 1.2 pmol/min per mm; *P* ≤ 0.05) (Figure 8, A and C). To determine whether there were any sex-related differences, we reanalyzed the data for males or females. CCDs from the male controls demonstrated a flow-induced increase in JNa (from 17.8 ± 4.9 to 52.3 ± 6.6 pmol/min per mm; *P* ≤ 0.01) and JK (from –0.4 ± 0.4 to –4.6 ± 0.5 pmol/min per mm; *P* ≤ 0.001) (Figure 8, B and D). CCDs from the female controls showed the same, as the increase in flow led to a significant increase in JNa (from 14.5 ± 3.2 to 47.5 ± 3.8 pmol/min per mm; *P* ≤ 0.05) and JK (from –0.9 ± 0.7 to –6.0 ± 1.3 pmol/min per mm; *P* ≤ 0.05) (Figure 8, B and D).

In CCDs from IC-*Piezo1*-KO mice of both sexes, JNa also increased in response to a 5-fold increase in luminal flow rate (9.7 ± 3.2 to 51.2 ± 5.2 pmol/min per mm; *P* ≤ 0.001); this was similar to that observed in control mice (ΔJNa KO = 42 ± 8 pmol/min per mm, vs. control = 34 ± 6 pmol/min per mm; *P* = 0.08) (Figure 8A). However, an increase in luminal flow rate failed to elicit an increase in JK in CCDs from IC-*Piezo1*-KO mice of both sexes (–0.5 ± 0.5 to 1.0 ± 1.2 pmol/min per mm; *P* = 0.08) (Figure 8C), indicative of a loss of the FIKS response. Analysis of sex-specific rates of transepithelial Na^+^ transport revealed once again that basal JNa and flow-stimulated JNa were not significantly different in male versus female IC-*Piezo1*-KO mice (Figure 8B), as the CCDs from male KOs showed an increase in JNa from 9.9 ± 4.1 to 49.4 ± 10.8 pmol/min per mm (*P* ≤ 0.05) whereas the CCDs from female KOs showed an increase in JNa from 9.4 ± 4.0 to 52.9 ± 2.3 pmol/min per mm (*P* ≤ 0.005). JK was unchanged in response to an increase in luminal flow rate in CCDs from KO males (–0.9 ± 0.5 to 1.3 ± 1.5 pmol/min per mm; *P* = 0.17) and KO females (–0.1 ± 0.1 to 0.6 ± 2.0 pmol/min per mm; *P* = 0.48), demonstrating the necessity of PIEZO1 in the FIKS response in isolated tubules from both sexes (Figure 8D).

### Deletion of Piezo1 alters acute K^+^ handling in male mice but not female mice.

**Figure 9:**
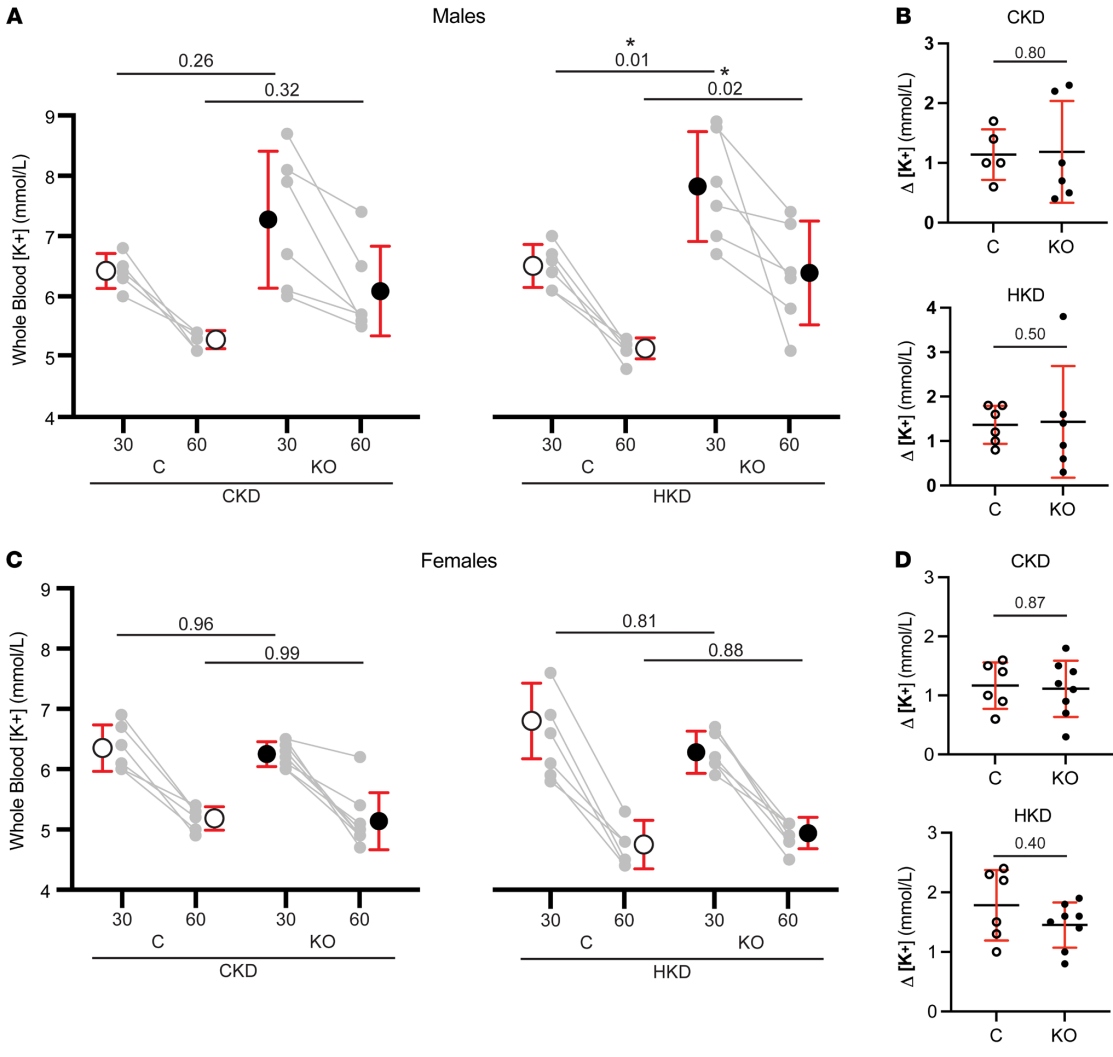
Male IC-*Piezo1*-KO mice have higher blood [K^+^] compared with control mice when challenged with an oral K^+^ load. Animals were gavage-fed with 150 μL of solution containing 5% KCl and 2% sucrose, and blood was sampled retroorbitally at 30 and 60 minutes after gavage. Males (**A**) and females (**C**) were fed a CK (left) or HK (right) diet for 10 days. For each dietary condition, control animals are shown on the left and KOs on the right. Individual points are shown in gray with a line connecting values from each individual animal. Summary data (mean ± SD) are shown on the outside of individual values for each time point. All groups displayed a decrease in blood [K^+^] at 60 minutes compared with the 30-minute blood draw, with calculated deltas shown for each group (**B** and **D**). Two-way ANOVA with Šidák’s multiple-comparison test (**A** and **C**) or Mann-Whitney tests (**B** and **D**) were performed to test for significance, defined as *P* < 0.05. All *P* values for comparisons between control and KO animals within each dietary condition/time point are displayed on the graphs. The *n* values were as follows: males: 5 CK controls, 6 CK KO, 6 HK controls, 6 HK KO; females: 6 CK controls, 8 CK KO, 4 HK controls, 5 HK KO.

Blood electrolytes and other parameters were assessed in littermate control and IC-*Piezo1*-KO mice maintained on a CK or HK diet for 10 days. The groups showed no significant differences in blood electrolytes, including [K^+^], [Na^+^], or [Cl^–^], as well as hematocrit, blood urea nitrogen, and aldosterone between control and IC-*Piezo1*-KO mice (Table 1). Given these findings, we examined blood electrolytes following an acute K^+^ bolus by gavage feeding of 0.1 mmol KCl to the mice (Figure 9). While an acute K^+^ load has been shown to be rapidly buffered by a redistribution of K^+^ from the extracellular to the intracellular space, recent studies have found that mice also exhibit a rapid (within 30–60 minutes) increase in urinary K^+^ excretion following an acute K^+^ bolus (51–53). An acute K^+^ load is associated with both a rapid dephosphorylation of Na^+^-Cl^–^ cotransporter (NCC), reducing its activity and increasing delivery of Na^+^ to downstream tubular segments in the ASDN (51, 52), and an increase in ENaC processing and surface expression that is dependent on mTORC2 (53).

While no significant differences were noted in animals maintained on a CK diet, male IC-*Piezo1*-KO mice fed an HK diet exhibited significantly higher blood [K^+^] at 30 and 60 minutes after KCl gavage, compared with control male mice (7.8 ± 0.9 mM vs. 6.5 ± 0.4 mM at 30 minutes, *n* = 6, *P* < 0.05; 6.4 ± 0.9 mM vs. 5.1 ± 0.2 mM at 60 minutes, *n* = 6, *P* < 0.05) (Figure 9A). In contrast, similar blood [K^+^] levels were seen 30 and 60 minutes after KCl gavage in female IC-*Piezo1*-KO mice as compared with control animals fed an HK diet (6.3 ± 0.4 mM vs. 6.8 ± 0.6 mM at 30 minutes, *n* = 5 and 4, *P* = 0.81; 4.9 ± 0.3 mM vs. 4.8 ± 0.4 mM at 60 minutes, *n* = 5 and 4, *P* = 0.88) (Figure 9C). While IC-*Piezo1*-KO males showed an increase in blood [K^+^] as compared with controls, the difference between the 30- and 60-minute time points was not significant in any of the groups compared (Figure 9, B and D).

**Figure 10:**
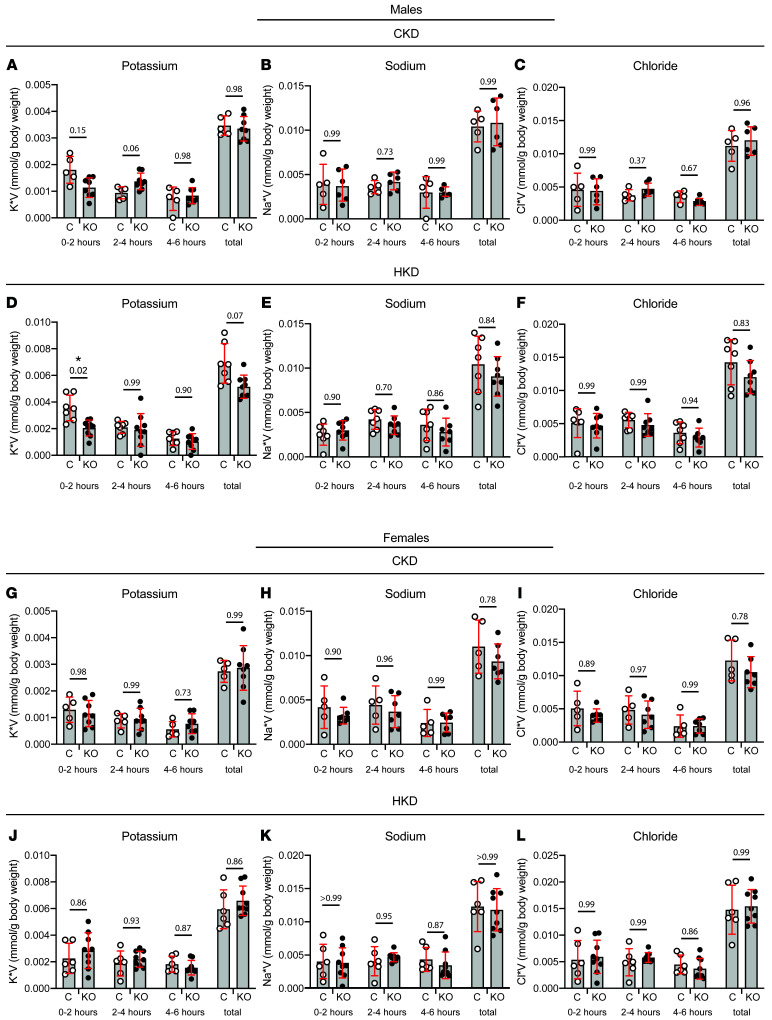
Male IC-*Piezo1*-KO mice have decreased urinary K^+^ excretion compared with control animals in the first 2 hours following a bolus saline injection. Male and female control and KO mice were weighed and given an intraperitoneal injection of normal saline equal to 10% of body weight. After injection, they were housed individually in wire-bottom metabolic cages to collect urine over 0–2, 2–4, and 4–6 hours. (**A**–**C**) Control and KO males maintained on CK diet had no difference in their urinary output of K^+^ (**A**), Na^+^ (**B**), or chloride (**C**). (**D**) However, when the males were retested after being given an HK diet for 10 days, KO mice displayed a significant decrease in their urinary K^+^ excretion within the first 2 hours after injection. (**E** and **F**) They displayed no difference in Na^+^ (**E**) or chloride (**F**) excretion. (**G**–**L**) Female control and KO animals had no differences in their excretion of K^+^ (**G**), Na^+^ (**H**), or chloride (**I**) on a CK diet or when the animals were fed an HK diet (**J**–**L**). Individual data points are shown with bar plots representing mean ± SD. Data were analyzed by 2-way ANOVA with a Greenhouse-Geisser correction to account for unequal variability of differences, followed by Šidák’s multiple-comparison test with significance being set at *P* < 0.05. *P* values are shown for the comparison between control and KO animals at each time point. The *n* values were as follows: males: 5 CK controls, 8 CK KO, 7 HK controls, 9 HK KO; females: 5 CK controls, 8 CK KO, 6 HK controls, 9 HK KO.

Urinary K^+^ excretion was examined after an intraperitoneal bolus injection of normal saline to enhance renal K^+^ secretion. IC-*Piezo1*-KO and control males and females maintained on CK diet showed similar rates of urinary excretion of K^+^, Na^+^, and Cl^–^ over the 6-hour collection period, except for a modest increase in urinary K^+^ excretion at 2–4 hours in KO males compared with controls (Figure 10). After the animals had been maintained on HK diet for 10 days, the male control animals excreted significantly more urinary K^+^ compared with the IC-*Piezo1*-KO males in the first 2 hours following injection of the bolus (3.6 ± 1.0 vs. 2.0 ± 0.60 μmol/g body weight, *n* = 7 control and 9 KO, *P* < 0.05). These animals displayed no significant difference in Na^+^ or Cl^–^ excretion when maintained on HK diet (Figure 10). HK-fed female animals also did not show significant differences in electrolyte excretion (Figure 10). Together, data collected in whole animals suggest that male, but not female, IC-*Piezo1*-KO mice fed an HK diet have an impaired K^+^ excretory response to an acute oral K^+^ load and to acute volume expansion.

### PIEZO1 regulates expression of BK channels in the CCD.

**Figure 11:**
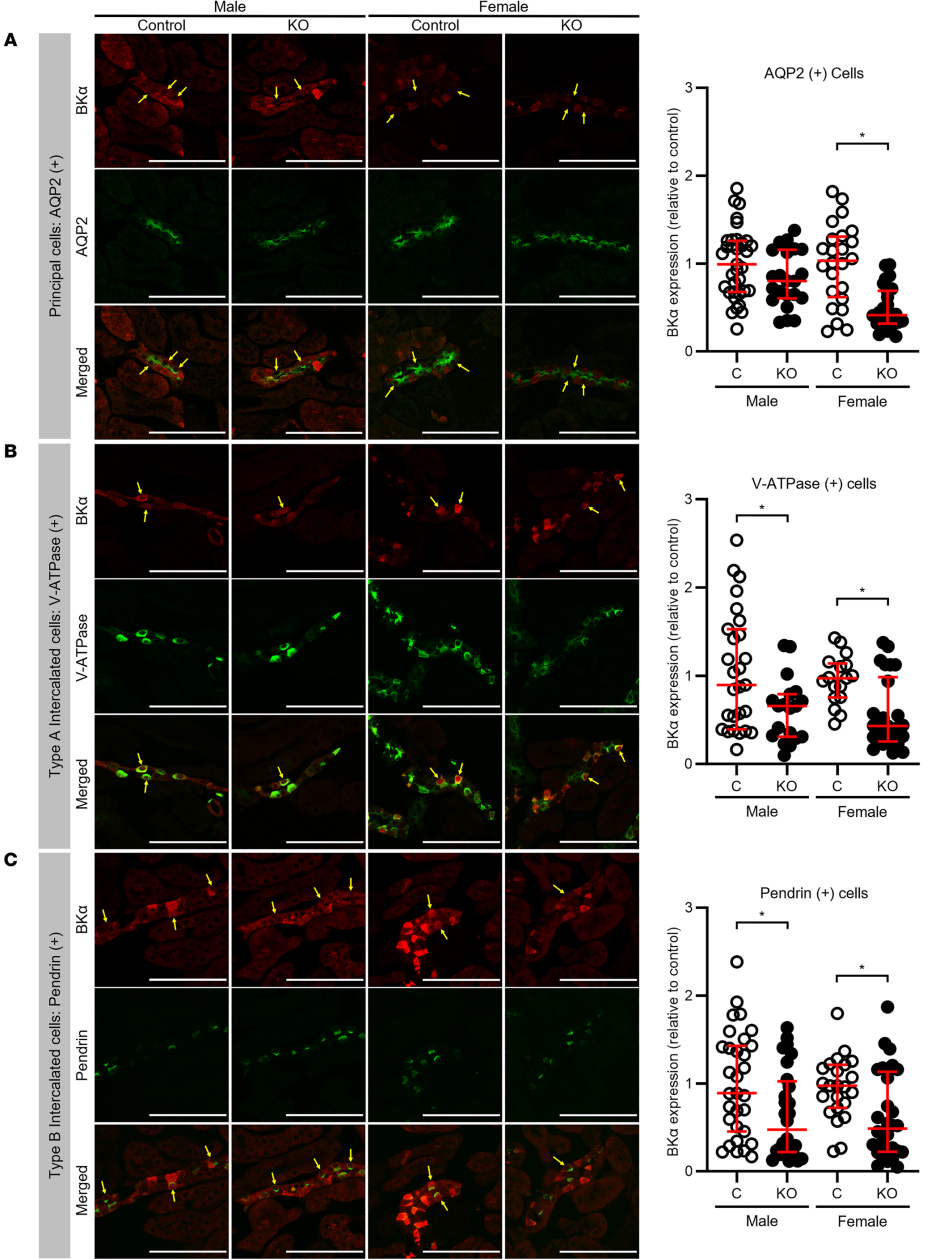
Expression of immunodetectable BKα subunit in HK-fed IC-*Piezo1*-KO mice compared with littermate controls. BKα subunit expression was assessed by immunofluorescence microscopy in type A and type B ICs and PCs in kidneys from KO and littermate control mice fed an HK diet (see Supplemental Methods). (**A**) Whole-cell expression of BKα subunit (red) was similar in PCs (identified by apical AQP2 staining; green) in KO (*n* = 22 cells) and control (*n* = 35 cells) male mice. However, whole-cell BKα expression was reduced in female KOs (*n* = 26 cells) versus controls (*n* = 24 cells). Median and interquartile values were compared using the Mann-Whitney rank sum test. *P ≤ 0.01 vs. control. (**B**) Whole-cell expression of BKα (red) was reduced in type A ICs (identified by apical V-ATPase staining; green) in male (*n* = 19 cells) and female (*n* = 26 cells) KO versus control mice of the same sex (*n* = 27 and 17 cells, respectively). Median and interquartile values were compared using the Mann-Whitney rank sum test. *P ≤ 0.05 vs. control. (**C**) Whole-cell expression of BKα was reduced in type B ICs (identified by apical pendrin staining; green) in male (*n* = 28 cells) and female (*n* = 28 cells) KO mice relative to control mice of the same sex (*n* = 31 and 23 cells, respectively). Median and interquartile values were compared using the Mann-Whitney rank sum test. *P ≤ 0.05 vs. control. Scale bars: 100 μm (**A**–**C**). Sections were analyzed from 3 male and 3 female control mice and 3 male and 3 female KO mice, all fed an HK diet for 10 days.

**Figure 12:**
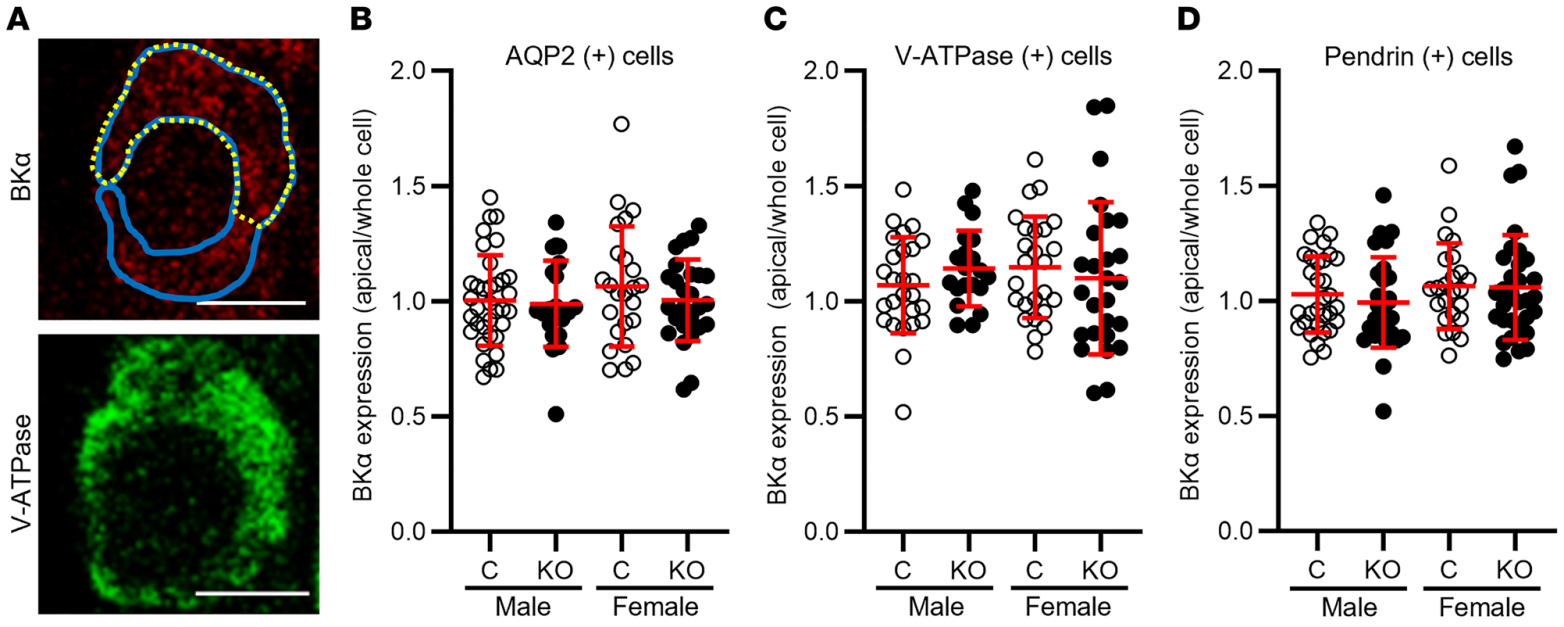
Relative apical to whole-cell BKα expression in ICs and PCs is not altered in IC-*Piezo1*-KO mice. BKα subunit expression was assessed by immunofluorescence microscopy in type A and type B ICs and PCs in kidneys from KO and littermate control mice fed an HK diet (see Supplemental Methods). (**A**) Example of regions quantified: whole cell (blue) and subapical plus apical (dotted yellow). Scale bars: 5 μm. (**B**–**D**) The ratio of subapical plus apical to whole-cell expression of BKα was similar in PCs (**B**) and type A (**C**) and type B (**D**) ICs of KO and control mice of both sexes. Data are mean ± SD; comparisons were made by 2-tailed *t* test. The number of cells for each group is indicated in the legend to Figure 11. Sections were analyzed from 3 male and 3 female control mice and 3 male and 3 female KO mice, all fed an HK diet for 10 days.

Given the absence of FIKS in IC-*Piezo1*-KO mice, we next assessed whether deletion of *Piezo1* led to changes in BKα subunit protein expression in CCDs. Kidney sections from littermate control and IC-*Piezo1*-KO mice fed an HK diet for 10 days were coimmunolabeled to detect the BKα subunit and either AQP2 to identify PCs, V-ATPase B1 subunit to identify type A ICs (apical B1 subunit staining), or pendrin to identify type B ICs. Quantification of the immunofluorescence signal revealed similar levels of BKα subunit whole-cell expression in AQP2-positive PCs in male control and IC-*Piezo1*-KO mice (Figure 11A), whereas its expression was reduced in female IC-*Piezo1*-KOs compared with controls. Whole-cell expression of BKα was significantly reduced in V-ATPase–positive type A ICs from both male and female IC-*Piezo1*-KO mice, compared with littermate controls (Figure 11B). In pendrin-positive type B ICs, whole-cell BKα expression was also significantly reduced in both male and female IC-*Piezo1*-KO mice relative to controls (Figure 11C). The ratio of subapical plus apical to whole-cell expression of BKα was similar in PCs, type A ICs, and type B ICs of male and female IC-*Piezo1*-KO and control mice (Figure 12).

## Discussion

We provide multiple lines of evidence that PIEZO1 functions as a mechanosensor in ICs, facilitating basolateral Ca^2+^ entry, triggering an increase in [Ca^2+^]_i_, and activation of BK channels. Our analyses of CCD IC cells in IC-*Piezo1*-KO mice demonstrate that PIEZO1 channels function as sensors of hydrodynamic forces, induced by increases in luminal flow rate, that are required for flow-dependent Ca^2+^ signaling and K^+^ secretion. Beyond showing that Ca^2+^ signaling and K^+^ secretion were inhibited by GsMTx4, and stimulated by Yoda1, the complete absence of a flow-induced increase in [Ca^2+^]_i_ in ICs from IC-*Piezo1*-KO mice and the absence of flow-induced K^+^ secretion (FIKS) in CCDs from these mice highlight the critical role of IC-expressed PIEZO1 in these processes. Somewhat surprisingly, we observed that the flow-induced elevation of [Ca^2+^]_i_ in the plateau phase was absent in IC-*Piezo1*-KO mice. This was unexpected, as the sustained plateau elevation in [Ca^2+^]_i_ has been proposed to reflect apical Ca^2+^ entry, mediated in part by TRPV4 (10–12). This observation would indicate that PIEZO1 acts upstream of the pathway that triggers TRPV4-mediated Ca^2+^ influx into the cell. Studies in pancreatic acinar and endothelial cells (54, 55) indicate that PIEZO1 induces the activation of PLA_2_ and subsequent activation of TRPV4 channels. PIEZO1 has also been shown to act upstream of TRPV4 to induce pathologic changes in endothelial cells due to shear stress (54). Specifically, in HUVECs and HEK293T cells expressing both PIEZO1 and TRPV4, high shear stress activates PIEZO1, leading to an initial increase in [Ca^2+^]_i_ that in turn triggers TRPV4 activation, and eventually to a loss of endothelial cell contacts, actin disruption, and endothelial cell monocyte adhesion.

An HK diet is known to enhance FIKS as well as BK channel activity and BKα subunit expression (1–4). We observed that an HK diet increases both PIEZO1 expression in ICs and PCs (Figure 4) and PIEZO1 functional activity, as demonstrated by Yoda1-dependent increases in [Ca^2+^]_i_ in ICs and well as PCs (Figure 2). In contrast, recent work by Pyrshev et al. did not observe an increase in PIEZO1 expression in response to an HK diet (although immunofluorescence was not quantified), and the authors did not examine changes in [Ca^2+^]_i_ in response to an increase in tubular flow (16). It is unclear why these potentially discrepant results were observed, and the mechanisms by which an HK diet increases PIEZO1 expression and activity (based on our observations) remain to be defined. In parallel, we found that disruption of *Piezo1* blunted BKα subunit expression in the ICs of mice on an HK diet (Figure 11), indicating that PIEZO1 expression or PIEZO1-dependent Ca^2+^ signaling has a role in enhancing BKα subunit expression. How this works is unknown, but PIEZO1 activation was previously linked to enhanced nuclear targeting of the Hippo signaling pathway yes-associated protein 1 (YAP) and WW domain–containing transcription regulator 1 (TAZ) (56, 57). Within the nucleus, YAP/TAZ can interact with the DNA-binding TEA domain family members (TEAD1–4) (58). In an analysis of genome-wide *Tead4* chromatin immunoprecipitation sequencing data sets of a variety of epithelial cancer cells, *KCNMA1* appears as a gene at which Tead4 binding was enriched (59). In addition, YAP/TAZ signaling has been shown to increase *Piezo1* transcription in squamous carcinoma cells (60), raising the possibility of a positive-feedback loop. While beyond the scope of this study, future investigation of the role of PIEZO1 activation in the transcriptional regulation of BK channels is warranted. We also noted reduced BKα subunit expression in PCs from female IC-*Piezo1*-KO mice on an HK diet. This was not observed in males, and we do not have an explanation for this finding.

Despite the loss of FIKS in IC-*Piezo1*-KO mice, we did not observe differences in blood [K^+^] and other electrolytes measured in littermate control and IC-*Piezo1*-KO mice, on either a CK or an HK diet when blood was analyzed in the morning (Table 1). We also did not observe differences in urinary electrolyte excretion over a 24-hour period (U_K_V and U_Na_V; Figure 10). To better assess differences in renal K^+^ handling, we examined changes in blood [K^+^] in response to acute K^+^ gavage feeding. In males on an HK diet, blood [K^+^] was significantly greater 30 and 60 minutes after gavage feeding in IC-*Piezo1*-KO mice, compared with controls (Figure 9). In contrast, differences in blood [K^+^] after gavage feeding were not observed in female mice on an HK diet, as well as in male and female mice on a control diet (Figure 9). We previously reported that mice that selectively lack either BKα subunit or long WNK1 expression in ICs have an increased blood [K^+^] when placed on an HK diet, a difference that was also only observed in males (2, 50). Other studies have also reported sex differences in renal K^+^ handling in rats on an HK diet (61). Together, these observations suggest that females may have different K^+^ excretory mechanisms that can compensate when they constitutively lack components of the FIKS response.

The complete absence of a flow-induced increase in [Ca^2+^]_i_ in ICs from IC-*Piezo1*-KO mice (Figure 6) was unexpected, based on the partial inhibition of the flow-induced [Ca^2+^]_i_ response detected in CCDs treated with the basolateral PIEZO1 inhibitor (Figure 1). Our discordant results may reflect incomplete inhibition of PIEZO1 by 5 μM GsMTx4. GsMTx4 does not bind directly to channel gating elements, but inhibits mechanosensitive channels by partitioning in and then altering the local membrane near the channel, and is sensitive to the local lipid environment (62).

We previously showed that PIEZO1 is also expressed in the basolateral membranes of PCs in the CCD (15), and we observed a reduction and increase in [Ca^2+^]_i_ in PCs in response to the PIEZO1 inhibitor GsMTx4 and activator Yoda1, respectively (Figures 1 and 2). While the role of PIEZO1 in PCs remains uncertain, it deserves investigation given that ENaC activity is altered in response to increases in [Ca^2+^]_i_, where both channel inhibition and activation have been reported (63, 64). Specifically, increases in [Ca^2+^]_i_ reduce ENaC activity, in part through Ca^2+^-dependent activation of calpain2 and cleavage of the scaffold protein MARCKS (myristoylated alanine-rich C kinase substrate), which blunts PIP_2_-dependent activation of ENaC (63). Others have suggested that increases in [Ca^2+^]_i_ activate ENaC (64). Future efforts will be targeted to generating a PC-specific *Piezo1*-KO mouse. Measurements of transepithelial transport in microperfused CCDs, channel currents by patch clamp in split open tubules, and whole-animal chemistries in PC-*Piezo1*-KO and littermate control mice should allow us to discern the functional role of PIEZO1 in PCs.

The preservation of flow-dependent activation of ENaC in CCDs from IC-*Piezo1*-KO mice is not a surprise, as previous work suggests that ENaC is directly activated by mechanical forces such as fluid shear stress (65–67). However, it is possible that changes in the flow-dependent release of paracrine factors from ICs influence net Na^+^ absorption in the ASDN. For example, PIEZO1 is critical for Ca^2+^-dependent ATP release, as has been described in RBCs (68–70), urothelial cells (46), and endothelial cells (26). Mechanical forces trigger release of autocrine/paracrine factors including ATP through the apical hemichannel connexin 30 and/or pannexin-1 channels, expressed in ICs (71, 72), and PGE_2_ (73), which may affect ENaC activity (74–76).

The apical central cilia of PCs and microvilli/microplicae of ICs, projecting into the urinary space where they can sense tubular fluid flow, have been proposed to have mechanosensory roles in renal tubular epithelia (77–80). However, the mechanosensors involved remain controversial (80, 81). Our studies reveal that despite its basolateral location, PIEZO1 also functions as a mechanosensor that is sensitive to luminal flow and the accompanying increase in tubular diameter. It is unknown whether there is functional interdependence between apical cilia and microvilli/microplicae and basolateral PIEZO1 channels. While likely not able to directly sense luminal fluid shear stress, a basolateral location of PIEZO1 is ideally suited to sense an increase in wall tension associated with increased tubule diameter due to an increase in tubular fluid flow and volume. We previously showed that increased flow is correlated with increased tubule diameter (9).

In summary, our results show that PIEZO1 functions as a mechanosensor in ICs (and possibly PCs) in the CCD, and that it is required for FIKS. While previous studies have suggested that PIEZO1 has a role in mechanosensing in isolated proximal tubule cells (41) and influences renal water handling in the distal nephron (40), our work is, to our knowledge, the first to demonstrate that PIEZO1 functions as a mechanosensor in the tubular epithelia lining the CCD and, by sensing wall tension in ICs, regulates transepithelial transport.

## Methods

Methods are available as Supplemental Methods (supplemental material available online with this article; https://doi.org/10.1172/JCI174806DS1).

### Study approval.

All animal breeding, housing, and protocols were approved by the Institutional Animal Care and Use Committees at the Icahn School of Medicine at Mount Sinai (ISMMS) and the University of Pittsburgh, facilities accredited by the Association for Assessment and Accreditation of Laboratory Animal Care International, and Zhengzhou University. Animals were euthanized in accordance with the National Institutes of Health *Guide for the Care and Use of Laboratory Animals* (National Academies Press, 2011).

### Data availability.

Values for all data points in the figures can be found in the Supporting Data Values file.

## Author contributions

LMS and TRK conceived and designed the study. RCG, SMM, FC, JS, MGD, GA, PW, WW, LMS, and TRK contributed to the acquisition, analysis, and/or interpretation of data for the work. RCG performed the assays in microperfused isolated tubules, including measurements of transepithelial transport and [Ca^2+^]_i_. SMM performed the whole-animal studies. All authors drafted the work or revised it critically for important intellectual content and approved the final version of the manuscript.

## Supplementary Material

Supplemental data

Supporting data values

## Figures and Tables

**Table 1:**
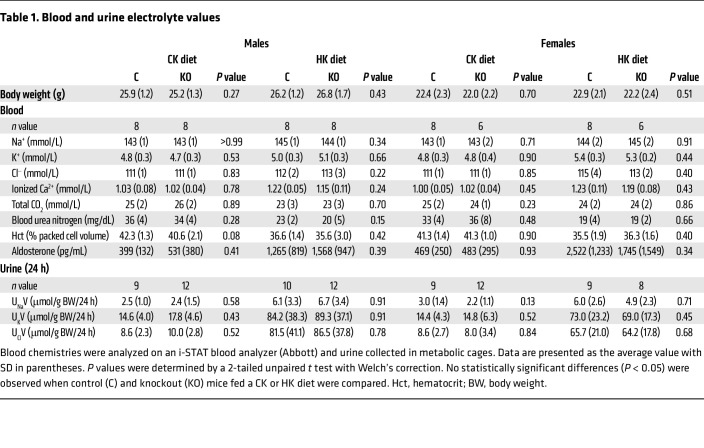
Blood and urine electrolyte values

## Version 1 03/01/2024

Electronic publication
