# Supplementary material for: PIEZO1 is a distal nephron mechanosensor and is required for flow-induced K+ secretion
Source: J Clin Invest. 2024 Mar 1;134(5):e174806. doi: 10.1172/JCI174806 (PMC10904061; doi:10.1172/JCI174806)
Supplement: Supplemental data [file jci-134-174806-s174.pdf]

Supplementary Material for

PIEZO1 is a distal nephron mechanosensor and is required for flow-induced K^+ secretion

Rolando Carrisoza-Gaytan[1*], Stephanie M. Mutchler[2*], Francisco Carattino[1], Joanne Soong[1], Marianela G. Dalghi[2], Peng Wu[3], WenHui Wang[4], Gerard Apodaca[2,5], Lisa M. Satlin[1], Thomas R. Kleyman[2,5,6]

[1]Department of Pediatrics, Icahn School of Medicine at Mount Sinai, New York, NY
[2]Department of Medicine, University of Pittsburgh, Pittsburgh, PA
[3]Department of Nephrology, The First Affiliated Hospital of Zhengzhou University, Zhengzhou, China
[4]Department of Pharmacology, New York Medical College, Valhalla, NY
[5]Department of Cell Biology, University of Pittsburgh, Pittsburgh, PA
[6]Department of Pharmacology and Chemical Biology, University of Pittsburgh, Pittsburgh, PA

The PDF file includes:
- Methods
- References for Methods

Methods

Animals

Mice were housed in standard caging under a 12:12-h day-night cycle and were allowed free access to tap water and chow. Mice were provided a 1% standard control K^+ (CK; TD.88238) diet at the time of weaning, and then switched for 10 days before study to either a 5.2% high K^+ (provided as KCl, HK; TD.09075) diet to maximally stimulate BK channel expression, or a <0.05% low K^+ (LK, TD.88239) diet, as indicated. All diets contained 0.3% Na^+ and were obtained from Envigo Teklad (Madison, WI).

Generation of intercalated cell-specific *Piezo1* KO (IC-*Piezo1*-KO) mice

Mice expressing Cre recombinase under the control of the promoter of the B1 subunit of the H^+-ATPase (B1-Cre) on a C57BL/6J background were used to generate an IC-specific KO. We have previously confirmed that the use of a B1-Cre confers an IC-specific KO (1).

Homozygous floxed *Piezo1* mice on a C57BL/6J background (*Piezo1*$^{flox/flox}$, Jackson Labs stock no. 029213), were bred with B1-Cre-expressing mice. Offspring hemizygous for B1-Cre and heterozygous for floxed *Piezo1* were bred with *Piezo1*$^{flox/flox}$ mice to obtain mice homozygous for floxed *Piezo1* and hemizygous for B1-Cre (termed IC-*Piezo1*-KO). Sex-matched *Piezo1*$^{flox/flox}$ littermates that did not express Cre were used as controls in all studies. Body weights of 12-16-week-old IC-*Piezo1*-KO and sex-matched littermate controls were similar for all studies. Mice of both sexes were used for experiments, unless indicated in the figure legend.

Genotyping was performed by PCR amplification using tail derived DNA. Floxed *Piezo1* gene expression was assessed with the following primers: (F) 5'-GCC TAG ATT CAC CTG GCT TC-3' and (R) 5'-GCT CTT AAC CAT TGA GCC ATC T-3'. The anticipated product size was ~380 bp in the KO, ~380 and 188 p in the heterozygotes, and 188 bp in the wild type. PCR

products were visualized after electrophoresis on a 1.5% agarose gel.

Quantification of *Piezo1* expression using fluorescent *in situ* hybridization (FISH)

Gene expression of *Piezo1* in ICs of the mouse kidney was analyzed using the RNAscope multiplex fluorescent kit (ACD; Newark, CA) and the following probes on fresh frozen samples: Mm-*Piezo1* in channel 1 (catalog #500511) and Mm-*Slc26a4* (pendrin) in channel 2 (catalog #452491). Briefly, mice were euthanized by CO_2 inhalation, followed by cervical dislocation, and their kidneys excised and washed with room temperature PBS buffer to remove excess blood. Kidneys were cut into halves in the axial plane, placed in a Tissue-Tek Cryomold (Sakura Finetek USA Inc, Torrance, CA) with Optimal Cutting Temperature embedding medium (OCT; Tissue Tek, Sakura Finetek USA Inc, Torrance, CA) and frozen by gentle contact of the bottom of the mold with liquid nitrogen (without submerging it). Samples were stored in a freezer at -80°C in sealed plastic bags until use. Sections (10 µm) were cut using a Leica CM1950 cryostat, collected on Superfrost™ Plus microscope slides (ThermoFisher Scientific, Waltham, MA) and stored at -80°C.

RNAscope protocol was performed as indicated by the ACD user's manual, with the following changes. Slides containing the sectioned tissue were removed from the -80°C freezer, and immediately fixed for 15 minutes at 4°C with neutral buffered formalin (4% w/v paraformaldehyde in 29.0 mM NaH_2PO_4 and 45.8 mM Na_2HPO_4) and treated with Protease IV for 5 minutes at room temperature. *Piezo1* and *Slc26a4* were developed using the TSA Plus Cyanine 3 (1:750; catalog #FP1170012UG Perkin Elmer) and TSA Plus Fluorescein (1:1000; catalog #FP1168015UG) reagents, respectively. Slides incubated with negative control probes (catalog #320871) to detect non-specific signal were run side-by-side with those treated with the probes and processed identically. Labeled tissue was mounted using ProLong Gold antifade mountant (Thermo Fischer), let dry overnight at room temperature in the dark, and subsequently stored at 4°C.

Images were captured using an HCX PLAN APO 40X oil objective (N.A. 1.25) attached to a DM6000B widefield microscope (Leica Microsystems, Buffalo Grove, IL) outfitted with a Gryphax Prokyon digital camera (Jenoptik). The multi-fluorescence capture mode of Gryphax software (Jenoptik) was used to collect images (1920 x 1200 pixels). For quantification, 6 random images of a kidney were taken for each animal. For each image, the total number of *Piezo1* fluorescent punctae associated with *Slc26a4*+ nuclei were counted and divided by the number of *Slc26a4*+ nuclei (dots/cell). For each animal, the average of the dots/cells values was calculated and constituted the level of *Piezo1* expression in *Scl26a4*+ cells for that particular mouse.

Electrophysiologic analyses of BK channel currents

CCDs were isolated as described above and split open. Perforated whole cell K^+ currents, recorded using an Axon 200A patch-clamp amplifier, were low-pass filtered at 1 KHz and digitized by an Axon interface (Digidata 1440A, Molecular Devices) (1, 2). Data were analyzed using the pCLAMP Software System 9 (Molecular Devices). Currents were measured using a bath solution containing (in mM): 130 K^+-gluconate, 10 KCl, 0.5 MgCl2, 1.5 $CaCl_2$, 10 HEPES (pH 7.4 with KOH). Yoda1 (5 µM) was added to the bath solution, as indicated, 10 min prior to initiation of patch clamp recordings. The pipette solution contained (in mM): 130 K^+-gluconate, 10 KCl, 1 $MgCl_2$, 8.72 $CaCl_2$, 10 EGTA (1 µM free Ca^{2+}), 5 HEPES (pH 7.4 with KOH). After forming a high-resistance seal (>2 GΩ), the membrane capacitance was monitored until the whole-cell patch configuration was formed. Currents were normalized to a membrane capacitance of 13 pF per cell. The cells were clamped at +60 mV and outward BK currents were identified by adding 100 nM charybdotoxin (ChTx, Tocris catalog # 1087) to the bath solution.

***In vitro* microperfusion of isolated CCDs**

In vitro microperfusion studies were performed using standard methodology, as previously described (1, 3) and briefly summarized as follows. A single kidney was immediately excised after death of the animal and coronal sections were placed in chilled dissection solution containing (in mM): 145 NaCl, 2.5 K_2HP0_4, 2.0 $CaCl_2$, 1.2 $MgS0_4$, 4.0 Na lactate, 1.0 Na citrate, 6.0 L-alanine, and 5.5 glucose (pH 7.4, 290±2 mOsm/kg). Single CCDs (0.2-0.4 mm length) were isolated by freehand dissection. A single tubule was studied from each mouse. Each isolated tubule was transferred to a temperature and O_2/CO_2-controlled specimen chamber, placed on the stage of an inverted microscope (Nikon Eclipse), mounted on concentric glass pipettes, and perfused and bathed at 37°C with Burg's solution containing (in mM): 120 NaCl, 25 $NaHCO_3$, 2.5 K_2HPO_4, 2.0 $CaCl_2$, 1.2 $MgSO_4$, 4.0 Na lactate, 1.0 Na_3 citrate, 6.0 L-alanine, and 5.5 D-glucose, pH 7.4, 290±2 mOsm/kg. During the 45 minute equilibration period and thereafter, the perfusion chamber was continuously suffused with a gas mixture of 95% O_2-5% CO_2 to maintain pH of the Burg's solution at 7.4 at 37°C. The bathing solution was continuously exchanged at a rate of 10 ml/h using a syringe pump (Razel, Stamford, CT).

As indicated, subsets of CCDs were treated with 5 µM *Grammostola spatulata* mechanotoxin #4 **(**GsMTx4, Tocris, catalog # 4912), a peptide inhibitor of cation selective mechanosensitive channels including PIEZO1 and 2 but not TRPV4 channels (4-8), or 1 or 5 µM Yoda1 ([1 (2-[5-[[(2,6-dichlorophenyl)methyl]thio]-1,3,4-thiadiazol-2-yl]pyrazine)], Tocris, catalog # 5586), a cell impermeant synthetic small molecule that acts as an agonist for both human and mouse PIEZO1 (9, 10). Yoda1 has no obvious effect on PIEZO2 (10).

Measurements of net transepithelial cation transport: After equilibration, three to four samples of tubular fluid were collected under water-saturated light mineral oil by timed filling of a calibrated ~10 nl volumetric constriction pipette under each experimental condition. Each tubule was perfused at both a slow (~0.9) and fast (~5.5 nl/min per mm) flow rate. The tubular flow rate was varied by adjusting the height of the perfusate reservoir. The sequence of flow

rates was randomized to minimize any bias induced by time-dependent changes in ion transport.

Finally, ouabain (200 µM) was added to the bath to inhibit all active transport and an additional three samples of tubular fluid were obtained for analysis to determine the composition of the solution delivered to the lumen of the tubule. Both [Na^+] and [K^+] of the perfusate and collected tubular fluid were determined by helium glow photometry, and the rates of net ion transport calculated using standard flux equations, as previously described (1, 11). As transport measurements were performed in the absence of transepithelial osmotic gradients and AVP, water transport was assumed to be zero. The calculated ion fluxes were averaged to obtain a single mean rate of transport for the tubule under each experimental condition.

Assessment of flow induced changes in [Ca^{2+}]$_i$: Isolated CCDs were transferred to the specimen chamber assembled with a No. 1 coverslip (Corning), painted with a 1 µl drop of poly-D-lysine hydrobromide 0.01% (BD Biosciences, Bedford, MA) and set on the stage of the Nikon Eclipse TE 300 inverted epifluorescence microscope linked to a Zyla 4.2 sCMOS camera (ANDOR Technology) interfaced with a digital imaging system (MetaFluor, Universal Imaging, Westchester, PA). Each microperfused CCD was positioned directly on the poly-D-lysine to immobilize the segment for the duration of the experiment. Following a 1 hour equilibration, CCDs were loaded with 20 µM of the acetoxymethyl ester of fura 2 (Calbiochem, La Jolla, CA) added to the bath for 20 min. The tubule was then rinsed with perfusate for 30 min. Fura 2-loaded CCDs were alternately excited at 340 nm and 380 nm and images, acquired every 3 sec, were digitized for subsequent analysis. After stable baseline 340 nm/380 nm fura 2 fluorescence intensity ratios (FIRs) were obtained at a slow flow rate, the luminal flow rate was increased acutely and FIRs were monitored, using our commercially available digital image analysis system (MetaFluor, Molecular Devices).

The mean FIRs, reflecting $[Ca^{2+}]_i$, for dull (identified as PCs) and bright (identified as ICs) cells, were calculated as were the changes in FIR from baseline at specified intervals of flow. Esterase-rich ICs can be distinguished from PCs in fura 2-loaded CCDs by their preferential accumulation of the indicator and brighter appearance when viewed under epifluorescence illumination (12, 13).

Localization and quantitation of PIEZO1 expression

Localization of PIEZO1 in PIEZO1-tandem dimer Tomato (tdT) reporter mice was performed as previously described (14). Fresh kidneys from mice fed a LK diet for 5 days or a CK or HK diet for 10 days were frozen directly in OCT. The protocol was performed as previously described with antibodies directed against AQP2 (SantaCruz SC9892 at a 1:700 dilution) and red fluorescent protein (Rockland 600-401-379, used at a dilution of 1:200 or 5.25 or 5.25 µg/ml). Quantification of PIEZO1-tdT fluorescence intensity in AQP2 negative ICs and AQP2 positive PCs was performed in ImageJ using the free hand selection tool to outline cells of interest.

Localization and quantitation of BKα expression

Kidneys from IC-*Piezo1*-KO mice and *Piezo1*$^{flox/flox}$ littermates fed a HK diet for 10 days were dissected, the capsule and poles discarded, and the remaining tissue immersed in 2.5% paraformaldehyde in phosphate-buffered saline (PBS containing 137 mM NaCl, 2.7 mM KCl, 8 mM Na_2HPO_4, and 2 mM KH_2PO_4, pH 7.4) overnight at 4°C and then cryoprotected by immersion in 30% sucrose in PBS with 0.02% azide. To examine BKα subunit localization within the cell, 20 µm thick cryosections were cut, pretreated with 10 mM sodium citrate buffer (90°C for 10 min) for antigen retrieval, rinsed with PBS, permeabilized with 0.3% Triton X-100 in PBS (10 min), and blocked with a solution containing 1% BSA, 10% fetal bovine serum, and 0.1% Triton X-100 in PBS at room temperature for 1 hr.

Sections were then incubated sequentially with: mouse IgG2a anti-BKα-subunit antibody (10 μg/mL, 75-408, Antibodies Incorporated) overnight at 4°C and A546 goat anti-mouse IgG2a (10 μg/mL, A21133, Thermo Fisher Scientific) for 1 hour at room temperature. To identify PCs and ICs, slices were stained with rat anti-AQP2 antibody (2.5 μg/mL, no. 20102rs, BiCell Scientific) followed by A488 goat anti-rat IgG secondary antibody (4 μg/mL, A11006, Thermo Fisher Scientific), rat anti-V-ATPase B1 subunit antibody (2.5 μg/mL, no. 20901, BiCell Scientific) followed by A488 goat anti-rat IgG secondary antibody (4 μg/mL, A11006, Thermo Fisher Scientific), or rabbit anti-pendrin antibody (5 μg/mL, no. 20501, BiCell Scientific) followed by A488 donkey anti-rabbit IgG (4 μg/mL, A21206, Thermo Fisher Scientific). After each antibody incubation, sections were rinsed three times for 10 minutes each at room temperature with PBS. Sections from 3 IC-*Piezo1*-KO and 3 control mice were immunostained concurrently under identical conditions.

Immunolabeled sections were mounted with SlowFade Diamond Antifade Mountant with DAPI (S36964, Thermo Fisher Scientific) and covered with a glass coverslip for imaging with a confocal laser scanning microscope (Leica TCS SP8 STED 3X, Leica Microsystems). Digital images (pinhole: 95.5 μm, step size: 1.0 μm) were collected from the cortical regions of the kidney section using a 63x oil immersion plan-Apochromat objective (numerical aperture: 1.4). The fluorescence intensity of apical and whole cell BKα ROIs was quantified using Leica Application Suite software (LAS X v. 3.7.5.24914, Leica Microsystems), as previously described (1), in PCs expressing AQP2, type A ICs expressing apical V-ATPase B1subunit, and type B ICs expressing apical pendrin.

Individually identified AQP2, V-ATPase, or pendrin positive cells in each tubule were selected for analysis by outlining the fluorescence signals in their apical and whole cell regions using the freehand tool of the software. For each outlined region, the sum of grayscale pixels/area outlined corresponding to the BKα subunit ROI was used to quantify fluorescence intensity for statistical analyses. Cells were analyzed from at least three different tubules per

kidney section. Fluorescence data for each cell type (PC, type A IC, or type B IC) was normalized to the average fluorescence intensity of that cell type in the male control.

Measurement of blood electrolytes and aldosterone

Mice were maintained on the CK or HK diet for 10 days were sacrificed under 2-5% isoflurane anesthesia. Blood was collected via cardiac puncture using a heparinized syringe. Whole blood electrolytes were measured using an i-STAT handheld analyzer (Abbott Point-of-Care, Inc., Princeton, NJ). Alternatively, mice maintained on the CK or HK diet for 10 days were gavage fed with 150 µl of a solution containing 5% KCl and 2% sucrose using a steel gavage needle attached to a syringe. Blood was drawn from the right retroorbital sinus 30 minutes post-gavage and from the left retroorbital sinus 60 minutes post-gavage using a heparinized glass capillary tube (Fisher 22-260-950) while the animals were lightly anesthetized with isothesia. Blood was analyzed on an i-STAT handheld analyzer (Abbott Point-of-Care, Inc., Princeton, NJ) using CHEM8+ cartridges.

In separate experiments, blood was taken from animals maintained on either CK or HK diet via retroorbital bleeds and spun down to separate plasma. The plasma was analyzed for aldosterone concentration using a commercially available ELISA run according to the manufacturer's instructions (Enzo Life Sciences).

Metabolic Cage Experiments

Animals were individually housed in wire-bottomed metabolic cages (Tecniplast) with access to water and gel food containing 1-2% agar mixed with ground commercial diet and water. Animals were allowed an acclimation period of at least 24 hours before any measurements were collected. For 24 hour electrolyte excretion, mice were allowed to reside in the cage undisturbed from 9 AM on day one to 9 AM the following day. Urine volume was measured and electrolyte content was analyzed via ion-specific electrodes (MedicaCorp). For studies examining K^+

secretion following a large volume load, animals were first encouraged to void by applying external pressure to the bladder. The animals were weighed and a bolus of normal saline equal to 10% of the body weight was given via intraperitoneal injection. Animals were returned to the metabolic cages and urine was collected in periods of 0-2 h, 2-4 h, and 4-6 h. All urines were analyzed for volume and electrolyte content, and the total of the 6 hour collection was summed from the individual values.

Statistics

Each experiment was performed using at least three mice per group. All results are expressed as mean±SD, unless otherwise indicated. An unpaired two-tailed Student's t-test was used to compare differences between floxed control and IC-*Piezo1*-KO mice, whereas a paired Student's t-test was used to detect differences between data collected in the same animal or CCD. The Mann-Whitney U test was used for analyses of data lacking a normal distribution. For multiple comparisons, a one-way ANOVA was used. For the metabolic cage data, each time point was analyzed separately with only the difference in output between control and IC-*Piezo1*-KO animals being compared as time was not a comparison of interest. Statistical significance was taken as $P < 0.05$.

References

1. Carrisoza-Gaytan R, Ray EC, Flores D, Marciszyn AL, Wu P, Liu L, et al. Intercalated cell BKalpha subunit is required for flow-induced K^+ secretion. *JCI Insight.* 2020;5(8):e130553.
2. Yue P, Zhang C, Lin DH, Sun P, and Wang WH. WNK4 inhibits Ca^{2+}-activated big-conductance potassium channels (BK) via mitogen-activated protein kinase-dependent pathway. *Biochim Biophys Acta.* 2013;1833(10):2101-10.
3. Ray EC, Carrisoza-Gaytan R, Al-Bataineh M, Marciszyn AL, Nkashama LJ, Chen JX, et al. L-WNK1 is required for BK channel activation in intercalated cells. *Am J Physiol-Renal.* 2021;321(2):F245-F54.
4. Miyamoto T, Mochizuki T, Nakagomi H, Kira S, Watanabe M, Takayama Y, et al. Functional role for Piezo1 in stretch-evoked Ca^{2+} influx and ATP release in urothelial cell cultures. *J Biol Chem.* 2014;289(23):16565-75.
5. Suchyna TM, Johnson JH, Hamer K, Leykam JF, Gage DA, Clemo HF, et al. Identification of a peptide toxin from Grammostola spatulata spider venom that blocks cation-selective stretch-activated channels. *J Gen Physiol.* 2000;115(5):583-98.
6. Bae C, Sachs F, and Gottlieb PA. The mechanosensitive ion channel Piezo1 is inhibited by the peptide GsMTx4. *Biochemistry.* 2011;50(29):6295-300.
7. Alcaino C, Knutson K, Gottlieb PA, Farrugia G, and Beyder A. Mechanosensitive ion channel Piezo2 is inhibited by D-GsMTx4. *Channels (Austin).* 2017;11(3):245-53.
8. Seghers F, Yerna X, Zanou N, Devuyst O, Vennekens R, Nilius B, et al. TRPV4 participates in pressure-induced inhibition of renin secretion by juxtaglomerular cells. *J Physiol.* 2016;594(24):7327-40.
9. Botello-Smith WM, Jiang W, Zhang H, Ozkan AD, Lin YC, Pham CN, et al. A mechanism for the activation of the mechanosensitive Piezo1 channel by the small molecule Yoda1. *Nat Commun.* 2019;10(1):4503.

10. Syeda R, Xu J, Dubin AE, Coste B, Mathur J, Huynh T, et al. Chemical activation of the mechanotransduction channel Piezo1. *Elife.* 2015;4:e07369.

11. Carrisoza-Gaytan R, Wang L, Schreck C, Kleyman TR, Wang WH, and Satlin LM. The mechanosensitive BKalpha/beta1 channel localizes to cilia of principal cells in rabbit cortical collecting duct (CCD). *Am J Physiol Renal Physiol.* 2017;312(1):F143-F56.

12. Liu W, Xu S, Woda C, Kim P, Weinbaum S, and Satlin LM. Effect of flow and stretch on the $[Ca^{2+}]_i$ response of principal and intercalated cells in cortical collecting duct. *Am J Physiol Renal Physiol.* 2003;285(5):F998-F1012.

13. Liu W, Murcia NS, Duan Y, Weinbaum S, Yoder BK, Schwiebert E, et al. Mechanoregulation of intracellular Ca^{2+} concentration is attenuated in collecting duct of monocilium-impaired orpk mice. *Am J Physiol Renal Physiol.* 2005;289(5):F978-88.

14. Dalghi MG, Clayton DR, Ruiz WG, Al-Bataineh MM, Satlin LM, Kleyman TR, et al. Expression and distribution of PIEZO1 in the mouse urinary tract. *Am J Physiol Renal Physiol.* 2019;317(2):F303-F21.
